# The mammalian faunas endemic to the Cerrado and the Caatinga

**DOI:** 10.3897/zookeys.644.10827

**Published:** 2017-01-10

**Authors:** Eliécer E. Gutiérrez, Jader Marinho-Filho

**Affiliations:** 1 PNPD Ecologia, Departamento de Zoologia, Universidade de Brasília, 70910-900 Brasília, DF, Brazil; 2 Division of Mammals, National Museum of Natural History, NHB 390, MRC 108, Smithsonian Institution, P.O. Box 37012, Washington DC 20013-7012, USA; 3 Departamento de Zoologia, Universidade de Brasília, 70910-900 Brasília, DF, Brazil

**Keywords:** Brazil, Bolivia, biogeography, checklist, conservation, Dry Diagonal, evolution, habitat, mammals, nomenclature, savannas, taxonomy

## Abstract

We undertook a comprehensive, critical review of literature concerning the distribution, conservation status, and taxonomy of species of mammals endemic to the Cerrado and the Caatinga, the two largest biomes of the South American Dry-Diagonal. We present species accounts and lists of species, which we built with criteria that, in our opinion, yielded results with increased scientific rigor relative to previously published lists – e.g., excluding nominal taxa whose statuses as species have been claimed only on the basis of unpublished data, incomplete taxonomic work, or weak evidence. For various taxa, we provided arguments regarding species distributions, conservation and taxonomic statuses previously lacking in the literature. Two major findings are worth highlighting. First, we unveil the existence of a group of species endemic to both the Cerrado and the Caatinga (i.e., present in both biomes and absent in all other biomes). From the biogeographic point of view, this group, herein referred to as Caatinga-Cerrado endemics, deserves attention as a unit – just as in case of the Caatinga-only and the Cerrado-only endemics. We present preliminary hypotheses on the origin of these three endemic faunas (Cerrado-only, Caatinga-only, and Caatinga-Cerrado endemics). Secondly, we discovered that a substantial portion of the endemic mammalian faunas of the Caatinga and the Cerrado faces risks of extinction that are unrecognized in the highly influential Red List of Threatened Species published by the International Union for Conservation of Nature (IUCN). “Data deficient” is a category that misrepresents the real risks of extinction of these species considering that (a) some of these species are known only from a handful of specimens collected in a single or a few localities long ago; (b) the Cerrado and the Caatinga have been sufficiently sampled to guarantee collection of additional specimens of these species if they were abundant; (c) natural habitats of the Cerrado and the Caatinga have been substantially altered or lost in recent decades. Failures either in the design of the IUCN criteria or in their application to assign categories of extinction risks represent an additional important threat to these endemic faunas because their real risks of extinctions become hidden. It is imperative to correct this situation, particularly considering that these species are associated to habitats that are experiencing fast transformation into areas for agriculture, at an unbearable cost for biodiversity.

## Introduction

The Caatinga and the Cerrado are the two largest biomes of the so-called Dry Diagonal of South America – a massive belt of land characterized by low precipitation and high seasonality – and together they occupy more than 30% of the Brazilian territory. The latter constitutes a vast (2 million km^2^) mosaic of xeromorphic vegetation types, from either dry (*campo limpo*) or humid (*campo úmido*) grasslands to woodlands (*cerradão*), and also harboring gallery forests, and patches of deciduous and semideciduous forests (Silva and Bates 2002, Voss et al. 2009, Carmignotto et al. 2012). The Cerrado encompasses areas from northeastern to southwestern Brazil, eastern Bolivia, and northern Paraguay. With deep soils rich in Aluminum, the Cerrado is irrigated by perennial watercourses (Coutinho 2006); its dry season lasts four months per year, with the rainfall concentrated in the wet season, and averaging annual values of 800–2000 mm (Ab’Sáber 1974, Nimer 1989). Neighboring the Cerrado is the Caatinga, a biome that occupies an extensive area (800,000 km^2^) of northeastern Brazil (Prado 2008). Its predominant habitat types are dry forests and xeric scrubs, although it also harbors patches of savannas (Andrade-Lima 1981). The climate of the Caatinga is markedly hot and dry, with highly erratic rainfall ranging from 240 to 1500 mm (Sampaio 1995, Prado 2008), and a severe period of drought lasting at least 5 months with high temperatures (Nimer 1989).

The Caatinga and the Cerrado harbor unique mammalian faunas whose evolutionary origin, biogeography, and conservation status remain poorly understood. Contrary to the mammalian fauna from open vegetation formations of northern South America (see Gutiérrez et al. 2014 and references therein), the mammals of the Caatinga and Cerrado biomes in northeastern, central, and southwestern South America have received some attention. New species either endemic to or predominantly distributed in these two biomes have been described (e.g., Sazima et al. 1978, Moojen et al. 1997, Bonvicino 2003, Bonvicino et al. 2003); data on the distribution of members of these faunas have been published (e.g., Marinho-Filho and Veríssimo 1997, Bezerra et al. 2007, 2010, Sá-Neto and Marinho-Filho 2013); patterns of phylogeographic variation have been detected (e.g., Carvalho et al. 2011, Nascimento et al. 2013); and assessments of their extinction risks have been conducted (e.g. Zimbres et al. 2012). In addition, a few studies have synthetized various kinds of ecological and biogeographic information of the mammalian faunas from the Caatinga and the Cerrado (Marinho-Filho et al. 2002, Oliveira et al. 2008, Carmignotto et al. 2012). Despite these valuable contributions, the study of these unique faunas remains in its infancy.

In order to facilitate further research, herein we provide lists and species accounts of the mammal species endemic to the Caatinga and the Cerrado. These species account focus on geographic distributions and conservation statuses according to published assessments. Due to the importance of clarifying taxonomic issues for biodiversity conservation (Gutiérrez and Helgen 2013), we provide discussions for taxa with unclear taxonomic statuses integrating all sources of relevant information currently available in the literature. We also conducted a critical discussion of the conservation status of our focal faunas. This aspect is particularly important taking into account that, due to expansion of land used for agriculture, only 47% of the original vegetation of the Cerrado remains (Beuchle et al. 2015), and its protected areas cover about 2% of this biome (Klink and Machado 2005), and that whereas the Caatinga preserves a larger portion of its original vegetation (63%; Beuchle et al. 2015), only ca. 1% of it is under strict federal protection, and it is being affected by accelerated desertification (Albuquerque et al. 2012).

## Methods

We conducted a critical review of literature concerning the distribution and conservation of mammalian species endemic to the Cerrado and the Caatinga. We used four main sources for constructing a preliminary list of focal species. These sources are the modern syntheses of the mammals of the Cerrado (Marinho-Filho et al. 2002) and the Caatinga (Oliveira et al. 2008), and their most recent update (Carmignotto et al. 2012); the most recent list of the mammals of Brazil (Paglia et al. 2012); and the two currently available volumes of the book series Mammals of South America (Gardner 2008, Patton et al. 2015). The preliminary list was then refined to obtain a final list by: (1) considering information from a plethora of studies published in peer-reviewed journals and books; (2) removing species for which no enough evidence of their nature as either endemic or valid species have been published – this applied also to cases in which authors mentioned the existence of unpublished data or manuscripts in support of their views, but such information remain unavailable for scientific scrutiny.

Two considerations regarding the scope of the present study need to be made. First, the geographic scope of our study differs from some of those used in previous studies. For example, contrary to Carmignotto et al. (2012), we did not regard the Pantanal as part of the Cerrado, as we consider it to be a biome itself, with particular influences from other humid biomes (see Werneck 2011 and references therein). Second, although differentiated, isolated populations that are often recognized as subspecies can be of importance from the conservation point of view, given the limited knowledge currently available about these faunas, we herein focused only on taxa recognizable at the species level.

We constructed species accounts composed primarily of two sections, distribution and conservation, but when necessary we also included an additional section devoted to taxonomic and nomenclatural considerations. For the distribution section, we indicated whether the species is endemic to the Cerrado, the Caatinga, or both, and presented a list of the administrative entities for which records supported by voucher specimens exist – with the only exceptions of *Cebus libidinosus* and *Lycalopex vetulus*, species for which some of the literature cited included ecological studies and direct observations made by mammalogists on free ranging individuals. We refer to publications that reported such records. We considered species as “endemic” in a strict way. That is, we excluded from our list of endemic species those that despite being predominantly distributed in the Cerrado, the Caatinga, or both, also occur in other biomes in areas that do not match patches of Caatinga or Cerrado vegetation. To do so, we followed the limits of the Brazilian biomes as defined by the *Instituto Brasileiro de Geografia e Estatística* (IBGE 2004a) and information reported in the literature regarding habitat types at collection localities. We did include in our list of endemics those species for which marginal records exist in the ecotones between our focal biomes (the Cerrado and the Caatinga) and other biomes, or in patches of Cerrado or Caatinga vegetation nearby the current contact zones between these and their neighbor biomes. Similarly, in cases in which a species was predominantly distributed in one of our focal biomes (e.g., the Caatinga) and transitional areas/ecotones with our other focal biome (i.e., the Cerrado, in this example), we considered that species endemic to the focal biome in which it is predominantly distributed (i.e. the Caatinga, in this example). In addition, we flag cases in which species have been regarded in the literature as endemics to our focal biomes, but that either more recent or revisited information demonstrate that they are not. We also flag the existence of species only known from transitional areas between our focal biomes and other biomes, as well as known undescribed species that might be endemic to our focal biomes, but for which further research is necessary to understand their distribution. In addition to the geographic distribution, for each species we indicate whether it has been recorded in open vegetation formations, forest, or both types of habitat.

For the conservation section, we used two sources: (1) the IUCN Red List of Threatened Species (available at http://www.iucnredlist.org), consulted in October–December 2016 (see citations relevant to each species under *Species accounts*); and (2) the official national list of threatened species published by the Brazilian government (ICMBIO-MMA 2016). The latter list only mentions species to which a category of threat has been assigned (i.e., it does not include species that are not threatened). We compared the conservation status (risk of extinction) that these two sources assigned to each species, and provided recommendations to improve their accuracy with regard to the focal species of this study.

## Results

### Species accounts

#### Didelphimorphia, Didelphidae, Didelphinae, Thylamyini

##### Cryptonanus agricolai

Taxon classificationAnimaliaDidelphimorphiaDidelphidae

(Moojen, 1943)

###### Distribution.

*Cryptonanus agricolai* is endemic to the Cerrado and the Caatinga, and has also been collected in contact zones between these biomes and the Amazon in northern Mato Grosso and southwestern Piauí states (Bezerra et al. 2009). Records of *Cryptonanus agricolai* in the Atlantic forest were obtained from pellets of *Tyto alba* and might actually correspond to individuals captured in the Cerrado, the Caatinga, or both (Souza et al. 2010), as these owls can potentially forage through long distances (up to ~31 km; Hegdal and Blaskiewicz 1984). According to Bezerra et al. (2014), unpublished molecular data suggest that *Cryptonanus agricolai* is absent from the Cerrado; however, we will refrain from adopting this view until these data become publicly available (contra Carmignotto et al. 2012). The current, known distribution of *Cryptonanus agricolai* includes the Brazilian states of Ceará, Goiás, Mato Grosso, Mato Grosso do Sul, Minas Gerais, Pernambuco, Piauí, Sergipe, and Tocantins (Voss et al. 2005, Bezerra et al. 2009, 2014, Carmignotto and Aires 2011, Bonvicino et al. 2012, Gómes et al. 2015, Gurgel-Filho et al. 2015, Hannibal and Neves-Godoi 2015, de la Sancha and D’Elía 2015). Alleged records from a locality in São Paulo state have been mentioned in the literature (see Martin et al. 2012), but they were made based on animals that were released (i.e., no voucher specimens are available) and identified using unreported criteria.

###### Conservation status.

The red list of the IUCN ver. 3.1 assigned the category “Data Deficient” to *Cryptonanus agricolai* (see Carmignotto et al. 2016a). The species was not included in the official list of threatened species of Brazil (ICMBio-MMA 2016).

##### Thylamys (Xerodelphis) karimii

Taxon classificationAnimaliaDidelphimorphiaDidelphidae

(Petter, 1968)

###### Distribution.

*Thylamys karimii* is endemic to the Cerrado and the Caatinga, and has been recorded in the Brazilian states of Bahia, Goiás, Mato Grosso, Minas Gerais, Pernambuco, Piauí, Rondônia, Sergipe, and Tocantins, and in the Distrito Federal (Carmignotto and Monfort 2006, Carvalho et al. 2009, Bruna et al. 2010, Carmignotto and Aires 2011, Bonvicino et al. 2012, Bezerra et al. 2014). The species has not been reported for Bolivia, but its presence on the Serranía de Huanchaca in the Santa Cruz department of that country might be expected (Giarla et al. 2010).

###### Conservation status.

The red list of the IUCN ver. 3.1 assigned the category “Vulnerable” to *Thylamys karimii* (see Carmignotto et al. 2016b). The species was not included in the official list of threatened species of Brazil (ICMBIO-MMA 2016).

##### Thylamys (Xerodelphis) velutinus

Taxon classificationAnimaliaDidelphimorphiaDidelphidae

(Wagner, 1842)

###### Distribution.

*Thylamys velutinus* is endemic to the Cerrado, and has been recorded in the Brazilian states of Bahia, Minas Gerais, São Paulo, and in the Distrito Federal (Vieira and Palma 1996, Bonvicino and Bezerra 2003, Giarla et al. 2010, Bonvicino et al. 2014). Marginal records are known from transitional areas that harbor isolated patches of Cerrado vegetation embedded within the Atlantic Forest biome (see Caceres 2012 and references therein).

###### Conservation status.

The red list of the IUCN ver. 3.1 assigned the category “Near Threatened” to *Thylamys velutinus* (see Carmignotto and Astúa 2016). The species appears in the official list of threatened species of Brazil with the category “Vulnerable” (ICMBIO-MMA 2016).

#### Cingulata, Dasypodidae, Tolypeutinae, Tolypeutini

##### Tolypeutes tricinctus

Taxon classificationAnimaliaCingulataDasypodidae

(Linnaeus, 1758)

###### Distribution.

*Tolypeutes tricinctus* is endemic to the Cerrado and the Caatinga (contra Wetzel et al. 2007), and has been recorded in the Brazilian states of Alagoas, Bahia, Ceará, Goiás, Maranhão, Paraíba, Pernambuco, Piauí, Sergipe, and Tocantins (Sanborn 1930, Moojen 1943; Silva and Oren 1993, Santos et al. 1994, Oliveira 1995, Marinho-Filho et al. 1997, Zimbres et al. 2012, 2013, Feijó et al. 2015a). The species had been traditionally considered an endemic of the Caatinga biome, however records of the species well within the Cerrado biome and in native Cerrado habitat type have been reported (Marinho-Filho et al. 1997; see also figure 2 in Feijó et al. 2015a).

###### Conservation status.

The red list of the IUCN ver. 3.1 assigned the category “Vulnerable” to *Tolypeutes tricinctus* (see Miranda et al. 2014). The species appears in the official list of threatened species of Brazil with the category “Endangered” (ICMBIO-MMA 2016).

#### Chiroptera, Phyllostomidae, Lonchophyllinae

##### Lonchophylla bokermanni

Taxon classificationAnimaliaChiropteraPhyllostomidae

Sazima, Vizotto & Taddei, 1978

###### Distribution.

*Lonchophylla bokermanni* is endemic to the Cerrado, where it has been collected in the Brazilian state of Minas Gerais (Dias et al. 2013, Teixeira et al. 2014, Almeida et al. 2016). Specimens collected in the Atlantic Forest of Brazil and previously identified as *Lonchophylla bokermanni* (by Nascimento et al. 2013) actually correspond to the recently described *Lonchophylla peracchii* (Dias et al. 2013).

###### Conservation.

The red list of the IUCN ver. 3.1 assigned the category “Endangered” to *Lonchophylla bokermanni* (see Aguiar et al. 2016; see also Teixeira et al. 2014). The species was not included in the official list of threatened species of Brazil (ICMBIO-MMA 2016).

##### Lonchophylla dekeyseri

Taxon classificationAnimaliaChiropteraPhyllostomidae

Taddei, Vizotto & Sazima, 1983

###### Distribution.

*Lonchophylla dekeyseri* is endemic to the Cerrado (contra Leal et al. 2013), and has been collected in the Brazilian states of Goiás, Mato Grosso do Sul, Minas Gerais, and in the Distrito Federal (Taddei et al. 1983, Coelho and Marinho-Filho 2002, Aguiar et al. 2014, Moratelli and Dias 2015, Almeida et al. 2016). Additional specimens assigned to *Lonchophylla dekeyseri* exist for the Bolivian state of Santa Cruz (in the Cerrado), and for the Brazilian states of Piauí (in the Cerrado-Caatinga ecotone) and Paraíba (in the Caatinga) (Woodman and Timm 2006, Leal et al. 2013). However, the taxonomic identifications of these specimens need to be reevaluated based on the morphological criteria recently proposed by Moratelli and Dias (2015), who noted the following: “*We are not convinced that Lonchophylla dekeyseri occurs in the Bolivian savannah and in the Cerrado–Caatinga ecotone in NE Brazil. One of the specimens supporting these records was examined a long time ago (DZSJRP 11459), and the other two (USNM 584472, 584473) are distinct from other samples of Lonchophylla dekeyseri as determined in a previous discriminant function analysis. These specimens are not included in this analysis because we were not able to compare them with samples from other localities.*” We provisionally regard *Lonchophylla dekeyseri* as endemic to the Brazilian Cerrado until further studies determine the taxonomic identity of specimens collected in Bolivia, the Cerrado-Caatinga ecotone, and the Caatinga.

###### Conservation status.

The red list of the IUCN ver. 3.1 assigned the category “Endangered” to *Lonchophylla dekeyseri* (see Aguiar and Bernard 2016). The species appears in the official list of threatened species of Brazil with the category “Endangered” (ICMBIO-MMA 2016).

##### Lonchophylla inexpectata

Taxon classificationAnimaliaChiropteraPhyllostomidae

Moratelli & Dias, 2015

###### Distribution.

*Lonchophylla inexpectata* is endemic to the Caatinga, and has been recorded in the Brazilian states of Pernambuco and Bahia (Moratelli and Dias 2015).

###### Conservation status.

The red list of the IUCN ver. 3.1 has not yet evaluated the extinction risk of *Lonchophylla inexpectata*. The species was not included in the official list of threatened species of Brazil (ICMBIO-MMA 2016). Confirmed specimens of *Lonchophylla inexpectata* are currently known from only three localities. Although an exhaustive revision of specimens housed in zoological collections might reveal a higher number of localities for the species, a provisional conservation status denoting some risk of extinction seems a sensible action considering the extremely low number of confirmed localities, particularly considering trends of habitat loss in the Caatinga (Albuquerque et al. 2012).

##### Xeronycteris vieirai

Taxon classificationAnimaliaChiropteraPhyllostomidae

Gregorin & Ditchfield, 2005

###### Distribution.

*Xeronycteris vieirai* is endemic to the Caatinga, and has been recorded in the Brazilian states of Bahia, Minas Gerais, Paraíba, and Pernambuco (Gregorin and Ditchfield 2005, Nogueira et al. 2007, 2014a, 2015, Astúa and Guerra 2008).

###### Conservation status.

The red list of the IUCN ver. 3.1 assigned the category “Data Deficient” to *Xeronycteris vieirai* (see Solari 2015). The species appears in the official list of threatened species of Brazil with the category “Vulnerable” (ICMBIO-MMA 2016). Eleven years after its description (Gregorin and Ditchfield 2005), *Xeronycteris vieirai* is known from only eight specimens and seven localities from the Caatinga (Gregorin and Ditchfield 2005, Nogueira et al. 2007, 2014a, 2015, Astúa and Guerra 2008), a biome with large disturbed areas as well as areas undergoing desertification (Albuquerque et al. 2012). Considering habitat loss, the specialized diet and the endemic nature of *Xeronycteris vieirai*, Gregorin and Ditchfield (2005) noted that the species might be one of most threatened species of mammals in Brazil. We agree with this view, and strongly encourage NGOs and governmental agencies in charge of producing influential “red lists of threatened species” to revaluate the conservation status they have assigned to *Xeronycteris vieirai*; a realistic category for the species should reflect at the very least a moderate risk of extinction, particularly considering trends of habitat loss in the Caatinga (Albuquerque et al. 2012).

#### Chiroptera, Phyllostomidae, Micronycterinae

##### Micronycteris sanborni

Taxon classificationAnimaliaChiropteraPhyllostomidae

Simmons, 1996

###### Distribution.

*Micronycteris sanborni* is endemic to the Cerrado and the Caatinga, and has been recorded in the Brazilian states of Ceará, Minas Gerais, Paraíba, Piauí, Pernambuco, and Tocantins (Simmons 1996, Gregorin et al. 2008, Cunha et al. 2009, Feijó et al. 2010, 2015b, Nogueira et al. 2015, Silva et al. 2015). An alleged record of the species for the Amazon biome has been recently published (López-Baucells et al. 2013), but no voucher specimen supports this claim and the individual that forms the basis of this record lacked the pure white ventral pelage coloration that seems to be characteristic of *Micronycteris sanborni* (see Simmons 1996, Feijó et al. 2015b). Applying Hitchens’ razor, we dismiss the alleged record for the Amazon biome (contra López-Baucells et al. 2013), a view we share with other authors (see Nogueira et al. 2014b, Feijó et al. 2015b). In addition, as noted by Feijó et al. (2015b), alleged records from the state of Mato Grosso do Sul need to be confirmed. One of the two specimens that formed the basis of these records (see Santos et al. 2010) lacks the pure white ventral pelage coloration typical of confirmed vouchers of *Micronycteris sanborni* (see above) – and according to Siles et al. (2013) it might actually correspond to *Micronycteris yatesi*. No information about the morphology of the other specimen has been published (see Cunha et al. 2009).

###### Conservation status.

The red list of the IUCN ver. 3.1 assigned the category “Data Deficient” to *Micronycteris sanborni* (see Tavares and Aguirre 2008). The species was not included in the official list of threatened species of Brazil (ICMBIO-MMA 2016).

#### Chiroptera, Phyllostomidae, Stenodermatinae

##### Chiroderma vizottoi

Taxon classificationAnimaliaChiropteraPhyllostomidae

Taddei & Lim, 2010

###### Distribution.

*Chiroderma vizottoi* is endemic to the Caatinga, and has been recorded in the Brazilian states of Ceará and Piauí (Gregorin et al. 2008, Taddei and Lim 2010, Silva et al. 2015).

###### Conservation status.

The red list of the IUCN ver. 3.1 has not yet evaluated the status of *Chiroderma vizottoi*. The species was not included in the official list of threatened species of Brazil (ICMBIO-MMA 2016).

#### Primates, Cebidae, Callitrichinae

##### Callithrix penicillata

Taxon classificationAnimaliaPrimatesCebidae

(É. Geoffroy in Humboldt, 1812)

###### Distribution.

*Callithrix penicillata* is endemic to the Cerrado, and has been recorded in the Brazilian states of Bahia, Goiás, Maranhão, Minas Gerais, Piauí, São Paulo, and in the Distrito Federal (de Vivo 1991, Miranda and Faria 2001, Rylands et al. 2009, Coimbra-Filho et al. 2006, Bruna et al. 2010, Vilela and Del-Claro 2011, Garbino 2015b). However, according to Rylands et al. (2009 p. 36) the records from Maranhão, which were reported by de Vivo (1991) based on skins deposited at the Museu Nacional (Rio de Janeiro), likely correspond to introductions of the species in areas far away from the native range of the species. Similarly, translocations of individuals out of their native range have been considered as a possible causal explanation for the existence of a hybrid zone between *Callithrix penicillata* and *Callithrix geoffroyi* in the Atlantic Forest (Fuzessy et al. 2014). Rylands et al. (2009) also commented that habitat transformation (with loss of forest) and introductions (resulting from misguided release of confiscated animals) have led *Callithrix penicillata* to occupy, and perhaps replace other species of *Callithrix*, at localities south and east of its native range.

###### Conservation status.

The red list of the IUCN ver. 3.1 assigned the category “Least Concern” to *Callithrix penicillata* (see Rylands and Mendes 2008). The species was not included in the official list of threatened species of Brazil (ICMBIO-MMA 2016).

#### Primates, Cebidae, Cebinae

##### Cebus (Sapajus) libidinosus

Taxon classificationAnimaliaPrimatesCebidae

Spix, 1823

###### Distribution.

*Cebus libidinosus* is endemic to the Cerrado and the Caatinga, and has been recorded in the Brazilian states of Bahia, Ceará, Goiás, Maranhão, Minas Gerais, Paraíba, Piauí, Pernambuco, Rio Grande do Norte, São Paulo, Tocantins, and Distrito Federal (Pontes et al. 2006, Vilela 2007, Canale et al. 2009, Bruna et al. 2010, Lynch-Alfaro et al. 2012a, 2014, Feijó and Langguth 2013, Fragaszy et al. 2013, Gomes et al. 2015, Mendes et al. 2015, Nova et al. 2015).

###### Nomenclature.

We regard *libidinosus* as a member of the genus *Cebus*, subgenus Sapajus, and advocate for the use of the name *Sapajus* at the subgenus-level (contra Lynch-Alfaro et al. 2012a, 2012b, 2014). The division between the gracile (untufted) and robust (tufted) groups of capuchin monkeys has been known for over a century (see Elliot 1913, Hershkovitz 1949, 1955), and the names *Cebus* and *Sapajus* have been applied to them at the subgenus-level, respectively (e.g., Silva-Junior 2001, Ferreira et al. 2009, Casado et al. 2010). Lynch-Alfaro et al. (2012a) recommended elevating *Sapajus* to the genus-level based on their estimated age for the split between the gracile and robust lineages of *Cebus* (95% highest posterior density = 4.21–7.86 Ma). This estimate resulted from the analysis of sequence data from two mitochondrial genes. Subsequently, Lynch-Alfaro et al. (2012b) summarized known morphological and behavioral differences between gracile and robust lineages, restated the gene-tree argument of Lynch-Alfaro et al. (2012a), and advocated for elevating *Sapajus* at the genus level; ‘expert opinions’ have been invoked to promote this view (Lynch-Alfaro et al. 2014). Although several NGOs, ecologists, and ethologists started to adopt this proposal, dissent exists (e.g., Rosenberger 2012, Feijó and Langguth 2013, this study). Clearly, the differences and phylogenetic split between the two lineages of capuchin monkeys should be recognized with Linnean nomenclature; however, the most suitable action to do so, at least provisionally, is to use *Sapajus* and *Cebus* as subgenera of *Cebus*. At least three reasons support this view, as follows (see also Voss et al. 2014, Garbino 2015a):

(1) Elevating *Sapajus* to the genus level is unnecessary, as it does not accomplish anything than using the name at the subgenus level could not.

(2) At least for now, using the age since the split between *Sapajus* and *Cebus* as an argument to elevate *Sapajus* at the genus level is flawed for two reasons. First, because the age of that split, as estimated by Lynch-Alfaro et al. (2012a), was inferred based on data from a single locus, it should be considered a preliminary one (i.e., a working hypothesis). Secondly, the artifactual current taxonomy of platyrrhine monkeys (see Rosenberger and Matthews 2008, Rosenberger 2012, Garbino 2015a and references therein) prevents sensible comparisons of age of splits among pairs of platyrrhine sister genera. This is a consequence of the wide, dogmatic acceptance of genera that have been proposed on the basis of criteria that are typically used to recognize species (at best) in most other groups of mammals. A recent example of this phenomenon is provided by the recent proposal to validate the name *Leontocebus* as a genus, to contain the *nigricollis* group of *Saguinus*, using as an argument the fact that it is sympatric with other tamarin lineage (Rylands et al. 2016) – note that this same unacceptable criterion (i.e., sympatry) and divergence-time have been used by Byrne et al. (2016) to propose the recognition of *Cheracebus* as a “genus” and without even considering the more sensible option of regarding *Cheracebus* as a subgenus of *Callicebus*; we herein propose to use *Cheracebus*

 at the subgenus level only. Clearly, the currently inflated taxonomy of New World primates should be fixed, and several proposed genera should be lumped into fewer ones. This process has already begun (e.g., Garbino 2015a, this study), but it is far from completion.

(3) Continuing to recognize the long established, monophyletic genus *Cebus*, and subgenera *Sapajus* and *Cebus* within it, allows for more efficient communication among scientists. First, the use of the genus-(subgenus)-species format (i.e. using the subgenus name, when pertinent) readily confers phylogenetic information. In this case, the genus name *Cebus* informs about the sister-taxon relationship between the subgenera *Sapajus* and *Cebus* – supported by a number of synapomorphies (see Lynch-Alfaro et al. 2012b) – whereas the subgenera names, *Sapajus* and *Cebus*, recognize the differences between the two lineages that underwent different evolutionary histories. Furthermore, association of the genus name *Cebus* with the species epithets of both linages of capuchin monkeys have existed for decades, and unnecessarily disrupting this association (by elevating *Sapajus* to the genus level) might pose difficulties in scientific communication, for example for literature searches or for merging data from public repositories (e.g., GenBank, Global Biodiversity Information Facility).

We take the opportunity to emphasize the importance of using the subgenus rank to preserve nomenclatural stability, similar to what have been recently done for other groups of mammals (Giarla et al. 2010, Voss et al. 2014, Díaz-Nieto et al. 2016, Teta et al. 2016). This aspect is especially important for New World primates, whose generic and alpha-level taxonomy should be rectified in the upcoming decades.

###### Conservation status.

The red list of the IUCN ver. 3.1 assigned the category “Least Concern” to Cebus (Sapajus) libidinosus (see Rylands and Kierulff 2015). The species was not included in the official list of threatened species of Brazil (ICMBIO-MMA 2016).

#### Primates, Pitheciidae, Callicebinae, Callicebini

##### Callicebus (Callicebus) barbarabrownae

Taxon classificationAnimaliaPrimatesPitheciidae

Hershkovitz, 1990

###### Distribution.

*Callicebus barbarabrownae* is endemic to the Caatinga, and has been recorded in the Brazilian states of Bahia and Sergipe (Hershkovitz 1990b, Marinho-Filho and Veríssimo 1997, Printes et al. 2011 and 2013, Marques et al. 2013, Corsini and Moura 2014).

###### Nomenclature.

We regard *barbarabrownae* as a member of the genus *Callicebus*, subgenus Callicebus. The arguments presented by Byrne et al. (2016) to propose splitting *Callicebus* (as traditionally understood) into different genera are flawed for the same reasons we have already discussed under the account of *Cebus libidinosus* (see above).

###### Conservation status.

The red list of the IUCN ver. 3.1 assigned the category “Critically Endangered” to *Callicebus barbarabrownae* (see Veiga et al. 2008). The species appears in the official list of threatened species of Brazil with the category “Critically Endangered” (ICMBIO-MMA 2016).

#### Carnivora, Canidae, Caninae, Canini

##### Lycalopex vetulus

Taxon classificationAnimaliaCarnivoraCanidae

(Lund, 1842)

###### Distribution.

*Lycalopex vetulus* is endemic to the Cerrado and the Caatinga, and has been recorded in the Brazilian states of Bahia, Ceará, Goiás, Mato Grosso, Mato Grosso do Sul, Minas Gerais, Piauí, São Paulo, and in the Distrito Federal (Costa and Courtenay 2003, Courtenay et al. 2006, Dalponte 2009, Bocchiglieri et al. 2010, Ribeiro et al. 2010, Olifiers and Delciellos 2013; see also Supplementary Material of Bubadué et al. 2016). The species has also been observed in the Brazilian state of Tocantins (Carmignotto and Aires 2011).

###### Conservation status.

The red list of the IUCN ver. 3.1 assigned the category “Least Concern” to *Lycalopex vetulus* (see Dalponte and Courtenay 2008). The species appears in the official list of threatened species of Brazil with the category “Vulnerable” (ICMBIO-MMA 2016).

#### Rodentia, Caviidae, Caviinae

##### Galea spixii

Taxon classificationAnimaliaRodentiaCaviidae

(Wagler, 1831)

###### Distribution.

*Galea spixii* is endemic to the Caatinga, and has been recorded in the Brazilian states of Alagoas, Bahia, Ceará, Minas Gerais, Pará, Pernambuco, São Paulo (Bezerra 2008, Norgueira et al. 2015, Dunnum 2015 and references therein).

###### Taxonomy.

Some authors have regarded *Galea spixii* and *Galea flavidens* as different species (Ellerman 1941, Bonvicino et al. 2005, Weksler and Bonvicino 2008a, Dunnum 2015); however the latter species has never been incorporated into a phylogenetic study based on molecular data (e.g., Dunnum and Salazar-Bravo 2010), and a modern morphological study that included all extant species in the genus did not find differences to distinguish *Galea flavidens* from *Galea spixii* (Bezerra 2008; but see Bonvicino et al. 2008). We follow Bezerra (2008) in treating *Galea flavidens* as a junior synonym of *Galea spixii*. The same author also found that populations currently referred to as *Galea spixii* actually might be composed by multiple valid species (Bezerra 2008).

###### Conservation status.

The red list of the IUCN ver. 3.1 assigned the category “Least Concern” to *Galea spixii* (see Catzeflis et al. 2016a). The species was not included in the official list of threatened species of Brazil (ICMBIO-MMA 2016).

#### Rodentia, Caviidae, Hydrochoerinae

##### Kerodon acrobata

Taxon classificationAnimaliaRodentiaCaviidae

Moojen, Locks & Langguth, 1997

###### Distribution.

*Kerodon acrobata* is endemic to the Cerrado, and has been recorded only in the Brazilian states of Goiás and Tocantins (Moojen et al. 1997, Bezerra et al. 2010, Zappes et al. 2014).

###### Conservation status.

The red list of the IUCN ver 3.1 assigned the category “Data Deficient” to *Kerodon acrobata* (see Roach 2016). The species appears in the official list of threatened species of Brazil with the category “Vulnerable” (ICMBIO-MMA 2016).

##### Kerodon rupestris

Taxon classificationAnimaliaRodentiaCaviidae

(Wied-Neuwied, 1820)

###### Distribution.

*Kerodon rupestris* is endemic to the Caatinga, and has been recorded in the Brazilian states of Alagoas, Bahia, Ceará, Minas Gerais, Paraiba, and Pernambuco (Moojen et al. 1997, Lessa et al. 2005, Dunnum 2015 and references therein). Indications of the species presence in the Cerrado (e.g., Marinho-Filho et al. 2002, Carmignotto et al. 2012) do not seem to be supported by voucher specimens.

###### Conservation status.

The red list of the IUCN ver 3.1 assigned the category “Least Concern” to *Kerodon rupestris* (see Catzeflis et al. 2016b). The species appears in the official list of threatened species of Brazil with the category “Vulnerable” (ICMBIO-MMA 2016).

#### Rodentia, Cricetidae, Sigmodontinae, Akodontini

##### Gyldenstolpia planaltensis

Taxon classificationAnimaliaRodentiaCricetidae

(Avila-Pires, 1972)

###### Distribution.

*Gyldenstolpia planaltensis* is endemic to the Cerrado, and has been recorded in the Brazilian state of Mato Grosso, and in the Distrito Federal (Bezerra et al. 2011, Pardiñas and Bezerra 2015).

###### Conservation status.

The red list of the IUCN ver. 3.1 has not yet attempted to evaluate the extinction risk of *Gyldenstolpia planaltensis*. The species appears in the official list of threatened species of Brazil with the category “Endangered” (ICMBIO-MMA 2016).

##### Juscelinomys candango

Taxon classificationAnimaliaRodentiaCricetidae

Moojen, 1965

###### Distribution.

*Juscelinomys candango* is endemic to the Cerrado, and is only known from its type locality in the Brazilian Distrito Federal (Moojen 1965, Emmons 2015).

###### Conservation status.

The red list of the IUCN ver. 3.1 assigned the category “Extinct” to *Juscelinomys candango* (see Leite and Patterson 2008). The species appears in the official list of threatened species of Brazil with the category “Critically Endangered (Likely Extinct)” (ICMBIO-MMA 2016).

##### Juscelinomys huanchacae

Taxon classificationAnimaliaRodentiaCricetidae

Emmons, 1999

###### Distribution.

*Juscelinomys huanchacae* is endemic to the Cerrado, and is only known from the Bolivian department of Santa Cruz (Emmons 2015).

###### Conservation status.

The red list of the IUCN ver. 3.1 assigned the category “Data Deficient” to *Juscelinomys huanchacae* (see Dunnum et al. 2008). Since the known records are restricted to Bolivian cerrado, the species was not considered to be included in the official list of threatened species of Brazil (ICMBIO-MMA 2016).

##### Oxymycterus delator

Taxon classificationAnimaliaRodentiaCricetidae

Thomas, 1903

###### Distribution.

*Oxymycterus delator* is endemic to the Cerrado and the Caatinga, and has been recorded in the Brazilian states of Bahia, Ceará, Goiás, Mato Grosso, Mato Grosso do Sul, Minas Gerais, Paraná, Piauí, São Paulo, and Tocantins, and in the Distrito Federal; and in the Paraguayan departments of Canindeyú and Paraguarí (Emmons 1999, Hoffmann et al. 2002, Andrade et al. 2004, Bonvicino et al. 2005, 2008, 2012, 2014, Bruna et al. 2010, Ribeiro et al. 2010, Carmignotto and Aires 2011, Owen 2013, Gurgel-Filho et al. 2015, Oliveira and Gonçalves 2015, Stumpp et al. 2016). It has been indicated that the species occurs also in the Chaco biome (Carmignotto et al. 2012), but we are not aware of reported specimens that support this notion. D’Elía et al. (2008) noted that in Paraguay the species is restricted to the Oriental Region (east of the Paraguay River), where areas with Chaco’s physiognomy are absent and, instead, isolated patches of Cerrado can be found (Owen 2013).

###### Conservation status.

The red list of the IUCN ver. 3.1 assigned the category “Least Concern” to *Oxymycterus delator* (see Patterson et al. 2008). The species was not included in the official list of threatened species of Brazil (ICMBIO-MMA 2016).

##### Thalpomys cerradensis

Taxon classificationAnimaliaRodentiaCricetidae

Hershkovitz, 1990

###### Distribution.

*Thalpomys cerradensis* is endemic to the Cerrado, and has been recorded in the Brazilian states of Bahia, Goiás, Mato Grosso, Tocantins, and in the Distrito Federal (Hershkovitz 1990a, Andrade et al. 2004, Gonçalves et al. 2006, Ribeiro et al. 2010, Carmignotto and Aires 2011, Bonvicino et al. 2012, Pardiñas and Teta 2015).

###### Conservation status.

The red list of the IUCN ver 3.1 assigned the category “Least Concern” to *Thalpomys cerradensis* (see Marinho-Filho et al. 2016a). The species appears in the official list of threatened species of Brazil with the category “Vulnerable” (ICMBIO-MMA 2016).

##### Thalpomys lasiotis

Taxon classificationAnimaliaRodentiaCricetidae

Thomas, 1916

###### Distribution.

*Thalpomys lasiotis* is endemic to the Cerrado, and has been recorded in the Brazilian states of Bahia, Minas Gerais, Rondônia, and São Paulo, and in the Distrito Federal (Hershkovitz 1990a, Ribeiro et al. 2010, 2011, Rocha et al. 2011a, Pardiñas and Teta 2015).

###### Conservation status.

The red list of the IUCN ver 3.1 assigned the category “Least Concern” to *Thalpomys lasiotis* (see Marinho-Filho et al. 2016b). The species appears in the official list of threatened species of Brazil with the category “Endangered” (ICMBIO-MMA 2016).

#### Rodentia, Cricetidae, Sigmodontinae, Oryzomyini

##### Cerradomys marinhus

Taxon classificationAnimaliaRodentiaCricetidae

(Bonvicino, 2003)

###### Distribution.

*Cerradomys marinhus* is endemic to the Cerrado, and has been recorded in the Brazilian states of Bahia and Minas Gerais (Carmignotto and Aires 2011, Percequillo 2015, Tavares et al. 2016).

###### Conservation status.

The red list of the IUCN ver. 3.1 assigned the category “Data Deficient” to *Cerradomys marinhus* (see Bonvicino and Percequillo 2008). The species was not included in the official list of threatened species of Brazil (ICMBIO-MMA 2016).

##### Euryoryzomys lamia

Taxon classificationAnimaliaRodentiaCricetidae

(Thomas, 1901)

###### Distribution.

*Euryoryzomys lamia* is endemic to the Cerrado, and has been recorded in the Brazilian states of Minas Gerais and Goiás (Prado and Percequillo 2013).

###### Conservation status.

The red list of the IUCN ver. 3.1 assigned the category “Endangered” to *Euryoryzomys lamia* (see Percequillo and Weksler 2008). The species appears in the official list of threatened species of Brazil with the category “Endangered” (ICMBIO-MMA 2016).

##### Microakodontomys transitorius

Taxon classificationAnimaliaRodentiaCricetidae

Hershkovitz, 1993

###### Distribution.

*Microakodontomys transitorius* is endemic to the Cerrado, and has been recorded in the Brazilian Distrito Federal (Hershkovitz 1993, Bonvicino et al. 2014, Paresque and Hanson 2015).

###### Conservation status.

The red list of the IUCN ver. 3.1 assigned the category “Endangered” to *Microakodontomys transitorius* (see Marinho-Filho and Vieira 2010). The species appears in the official list of threatened species of Brazil with the category “Endangered” (ICMBIO-MMA 2016).

##### Oecomys cleberi

Taxon classificationAnimaliaRodentiaCricetidae

Locks, 1981

###### Distribution.

*Oecomys cleberi* is endemic to the Cerrado, and has been recorded in the Brazilian Distrito Federal (Locks 1981, Rocha et al. 2012, Carleton and Musser 2015).

###### Conservation status.

The red list of the IUCN ver. 3.1 assigned the category “Data Deficient” to *Oecomys cleberi* (see Costa et al. 2008). The species was not included in the official list of threatened species of Brazil (ICMBIO-MMA 2016).

##### Oligoryzomys moojeni

Taxon classificationAnimaliaRodentiaCricetidae

Weksler & Bonvicino, 2005

###### Distribution.

*Oligoryzomys moojeni* is endemic to the Cerrado, and has been recorded in the Brazilian states of Goiás and Tocantins (Miranda et al. 2009, Weksler and Bonvicino 2005, Carmignotto and Aires 2011, Di-Nizo et al. 2015, Gomes et al. 2015).

###### Conservation status.

The red list of the IUCN ver. 3.1 assigned the category “Data Deficient” to *Oligoryzomys moojeni* (see Weksler and Bonvicino 2008b). The species was not included in the official list of threatened species of Brazil (ICMBIO-MMA 2016).

##### Oligoryzomys rupestris

Taxon classificationAnimaliaRodentiaCricetidae

Weksler & Bonvicino, 2005

###### Distribution.

*Oligoryzomys rupestris* is endemic to the Cerrado, and has been recorded in the Brazilian states of Bahia, Goiás, and Minas Gerais (Weksler and Bonvicino 2005, Pereira and Geise 2009, Di-Nizo et al. 2015).

###### Conservation status.

The red list of the IUCN ver. 3.1 assigned the category “Data Deficient” to *Oligoryzomys rupestris* (see Weksler and Bonvicino 2008c). The species appears in the official list of threatened species of Brazil with the category “Endangered” (ICMBIO-MMA 2016).

##### Oligoryzomys stramineus

Taxon classificationAnimaliaRodentiaCricetidae

Bonvicino & Weksler, 1998

###### Distribution.

*Oligoryzomys stramineus* is endemic to the Cerrado and the Caatinga, and has been recorded in the Brazilian states of Bahia, Ceará, Goiás, Minas Gerais, Paraíba, Pernambuco and Piauí (Bonvicino and Weksler 1998, Weksler and Bonvicino 2005, 2015, Geise et al. 2010, Fernandes et al. 2012).

###### Conservation status.

The red list of the IUCN ver. 3.1 assigned the category “Least Concern” to *Oligoryzomys stramineus* (see Weksler and Bonvicino 2008d). The species was not included in the official list of threatened species of Brazil (ICMBIO-MMA 2016).

#### Rodentia, Cricetidae, Sigmodontinae, Phyllotini

##### Calassomys apicalis

Taxon classificationAnimaliaRodentiaCricetidae

Pardiñas, Lessa, Salazar-Bravo & Câmara, 2014

###### Distribution.

*Calassomys apicalis* is endemic to the Cerrado, and has been recorded only in the Brazilian state of Minas Gerais (Pardiñas et al. 2014).

###### Conservation status.

The red list of the IUCN ver. 3.1 has not yet attempted to evaluate the extinction risk of *Calassomys apicalis*. The species was not included in the official list of threatened species of Brazil (ICMBIO-MMA 2016).

##### Calomys expulsus

Taxon classificationAnimaliaRodentiaCricetidae

(Lund, 1840)

###### Distribution.

*Calomys expulsus* is endemic to the Cerrado and the Caatinga (contra Gurgel-Filho et al. 2015). All but one known records attributable to this species are located within these biomes; the exceptional record comes from a site likely harboring transitional conditions, in terms of physiognomy and climate, between those of the Caatinga and Atlantic Forest, in the Brazilian state of Pernambuco (see Gurgel-Filho et al. 2015, who referred to this species as *Calomys mattevii*, which we consider a junior synonym of *Calomys expulsus*; see “Taxonomy”, below). Specimens attributed to *Calomys expulsus* have been recorded in the Brazilian states of Bahia, Goiás, Minas Gerais, Pernambuco, Piauí, Sergipe, and in the Distrito Federal (Bonvicino and Almeida 2000, Bonvicino et al. 2003, Almeida et al. 2007, Haag et al. 2007, Bonvicino et al. 2012, Bezerra et al. 2014, Gurgel-Filho et al. 2015 and references therein, Nogueira et al. 2015). According to Gurgel-Filho et al. (2015), unpublished results of a phylogenetic study recovered samples of “*Calomys mattevii*” (=*Calomys expulsus*) in a clade in which samples from the states of Ceará and Tocantins – for which we presume no karyotype were available – were also included.

###### Taxonomy.

We provisionally consider the recently described *Calomys mattevii* as a junior synonym of *Calomys expulsus*. Gurgel-Filho et al. (2015) asserted that two specimens karyotyped by Geise et al. (1996), collected in Lagoa Santa (the type locality of *Calomys expulsus*), Minas Gerais, with 2n=36/FN=66, correspond to *Calomys expulsus*; however, Gurgel-Filho et al. (2015) did not examine these specimens. These authors alleged that the karyotype 2n=66/FN=68, widely attributed by authors to *Calomys expulsus* (e.g., Bonvicino and Almeida 2000, Mattevi et al. 2005, Haag et al. 2007, Bezerra et al. 2014, Salazar-Bravo 2015), would have to correspond to a different species (other than *Calomys expulsus*), which they described as *Calomys mattevii*. Although it is plausible that the specimens reported by Geise et al. (1996) were indeed *Calomys expulsus*, this cannot be assumed as certain because multiple species of *Calomys* might occur in Lagoa Santa (not only *Calomys expulsus*). In fact, Lagoa Santa is also the type locality of *Calomys tener* (see Winge 1887, Salazar-Bravo 2015), and the identity of the specimens from Lagoa Santa that were the basis of Geise et al.’s (1996) report has also been attributed to *Calomys cerqueirai* (Bonvicino et al. 2010; see also Colombi and Fagundes 2015, Salazar-Bravo 2015 and citations therein). Examination of the specimens from which Geise et al. (1996) obtained the reported karyotypes, and comparisons of their morphologies with respect to type material of *Calomys expulsus*, would be necessary to evaluate whether Gurgel-Filho et al.’s (2015) conjecture is correct. Unfortunately, it is unclear whether the specimens karyotyped by Geise et al. (1996) were ever deposited in a zoological collection, and whether their morphology matches that of *Calomys expulsus* – see similar concern expressed by Bonvicino and Almeida (2000, p. 347). Moreover, the karyotype reported by Geise et al. (1996) was not ever part of a formal peer-reviewed publication; it was rather reported as an abstract from a presentation at a scientific meeting – the 42th National Congress of Genetics of the *Sociedade Brasileira de Genética* – that have permeated into the literature. In addition, to support their conjecture and decision to describe *Calomys mattevii*, Gurgel-Filho et al. (2015) disregarded the use of morphological data as relevant to assess the taxonomic status of *Calomys mattevii*. However, we argue that it is possible to use morphometrics to discriminate among some species of *Calomys*; albeit not an easy task, and might not always lead to evidence that allow unambiguous discrimination among the studied species, several authors have accomplished it in the past (e.g., Bonvicino and Almeida 2000, Bonvicino et al. 2003, Cordeiro-Estrela et al. 2006).

In summary, given the aforementioned uncertainties in the description of *Calomys mattevii*, we provisionally consider it a junior synonym of *Calomys expulsus*. Collecting, karyotyping, and sequencing *Calomys* from the type locality of *Calomys expulsus* (see above), and using this material in comparative analyses that should include typical, or at least topotypical, material of other members of the large-size group of the genus (i.e., *Calomys callidus*, *Calomys callosus*, *Calomys cerqueirai*, and *Calomys tocantinsi*) is a necessary step to evaluate the taxonomic status of *Calomys mattevii*. On the meantime, we consider that karyotype 2n=66/FN=68 corresponds to *Calomys expulsus*, following Bonvicino and Almeida (2000), who asserted that the morphology of their karyotyped specimens is congruent with morphological characters and measurements of the holotype of *Calomys expulsus* as reported by Winge (1887) and Hershkovitz (1962), respectively.

###### Conservation status.

The red list of the IUCN ver 3.1 assigned the category “Least Concern” to *Calomys expulsus* (see Bonvicino and Geise 2008a). The species was not included in the official list of threatened species of Brazil (ICMBIO-MMA 2016).

##### Calomys tocantinsi

Taxon classificationAnimaliaRodentiaCricetidae

Bonvicino, Lima & Almeida, 2003

###### Distribution.

*Calomys tocantinsi* is endemic to the Cerrado, and has been recorded in the Brazilian states of Mato Grosso and Tocantins (Bonvicino et al. 2003, 2010, Cordeiro-Estrela et al. 2006, Rocha et al. 2011a).

###### Conservation status.

The red list of the IUCN ver. 3.1 assigned the category “Least Concern” to *Calomys tocantinsi* (see Bonvicino and Marinho-Filho 2008a). The species was not included in the official list of threatened species of Brazil (ICMBIO-MMA 2016).

#### Rodentia, Cricetidae, Sigmodontinae, Thomasomyini

##### Rhipidomys cariri

Taxon classificationAnimaliaRodentiaCricetidae

Tribe, 2005

###### Distribution.

*Rhipidomys cariri* is endemic to the Caatinga, and has been recorded in the Brazilian states of Bahia, Ceará, and Pernambuco (Tribe 2005, 2015, Geise et al. 2010, Fernandes-Ferreira et al. 2015, Gurgel-Filho et al. 2015).

###### Conservation status.

The red list of the IUCN ver. 3.1 assigned the category “Data Deficient” to *Rhipidomys cariri* (see Patton et al. 2008a). The species appears in the official list of threatened species of Brazil with the category “Vulnerable” (ICMBIO-MMA 2016).

#### Rodentia, Cricetidae, Sigmodontinae, Wiedomyini

##### Wiedomys cerradensis

Taxon classificationAnimaliaRodentiaCricetidae

Gonçalves, Almeida & Bonvicino, 2005

###### Distribution.

*Wiedomys cerradensis* is endemic to the Cerrado, and has been recorded in the Brazilian states of Bahia, Ceará, Goiás, and Tocantins (Gonçalves et al. 2005, Bezerra et al. 2013, Bonvicino 2015, Gurgel-Filho et al. 2015). Gurgel-Filho et al. (2015) indicated that the species also occurs in the Brazilian states of Piauí, Maranhão, Paraíba, and Pernambuco, but did not provide evidence supporting this assertion.

###### Conservation status.

The red list of the IUCN ver. 3.1 assigned the category “Data Deficient” to *Wiedomys cerradensis* (see Bonvicino and Marinho-Filho 2008b). The species was not included in the official list of threatened species of Brazil (ICMBIO-MMA 2016).

##### Wiedomys pyrrhorhinos

Taxon classificationAnimaliaRodentiaCricetidae

(Wied-Neuwied, 1821)

###### Distribution.

*Wiedomys pyrrhorhinos* is endemic to the Caatinga, and has been recorded in the Brazilian states of Alagoas, Bahia, Minas Gerais, Paraíba, and Pernambuco (Maia and Langguth 1987, Pereira and Geise 2009, Souza et al. 2011a, Bonvicino 2015, Nogueira et al. 2015). Bonvicino (2015) indicated that the species is also present in the state of Sergipe, but this assertion is based on an abstract presented at the XXIV Jornadas Argentinas de Mastozoología. Citation of this abstract is as follows:

Souza ALG, Pessôa LM, Menezes AN, Bezerra AMR, Bonvicino CR (2011) O rio São Francisco como provável barreira geográfica para as duas espécies do gênero *Wiedomys* (Rodentia). Revista del Museo de La Plata, Zoología 18(172): 163R. [abstract presented in the XXIV Jornadas Argentinas de Mastozoología, La Plata, Argentina, November 8–11, 2011].

###### Conservation status.

The red list of the IUCN ver. 3.1 assigned the category “Least Concern” to *Wiedomys pyrrhorhinos* (see Marinho-Filho and Langguth 2016). The species was not included in the official list of threatened species of Brazil (ICMBIO-MMA 2016).

#### Rodentia, Echimyidae, Eumysopinae

##### Carterodon sulcidens

Taxon classificationAnimaliaRodentiaEchimyidae

(Lund, 1838)

###### Distribution.

*Carterodon sulcidens* is endemic to the Cerrado, and has been recorded in the Brazilian states of Goiás, Mato Grosso, Mato Grosso do Sul, Minas Gerais, and in the Distrito Federal (Ribeiro et al. 2010, Bezerra et al. 2011, Bezerra and Bonvicino 2015a and references therein).

###### Conservation status.

The red list of the IUCN ver. 3.1 assigned the category “Data Deficient” to *Carterodon sulcidens* (see Lacher 2016). The species was not included in the official list of threatened species of Brazil (ICMBIO-MMA 2016).

##### Phyllomys brasiliensis

Taxon classificationAnimaliaRodentiaEchimyidae

Lund, 1840

###### Distribution.

*Phyllomys brasiliensis* is endemic to the Cerrado, and has been recorded in the Brazilian state of Minas Gerais (Emmons et al. 2002, Leite 2003, Leite and Loss 2015).

###### Conservation.

The red list of the IUCN ver. 3.1 assigned the category “Endangered” to *Phyllomys brasiliensis* (see Loss and Leite 2016). The species appears in the official list of threatened species of Brazil with the category “Endangered” (ICMBIO-MMA 2016).

##### Thrichomys apereoides

Taxon classificationAnimaliaRodentiaEchimyidae

(Lund, 1839)

###### Distribution.

*Thrichomys apereoides* is endemic to the Cerrado (contra Oliveira and Langguth 2004, Paglia et al. 2012), and has been recorded in the Brazilian state of Minas Gerais (Nogueira et al. 2015, Pessôa et al. 2015, Stumpp et al. 2016; but see “Taxonomy”, below).

###### Taxonomy.

Based on karyological data, Bonvicino et al. (2002) changed the traditional notion that the genus *Thrichomys* was monotypic (*Thrichomys apereoides*). Subsequently, two other studies gave support to the polytypic nature of *Thrichomys*, one based on karyological data and morphometric analyses (Pessôa et al. 2004), and the other on karyological data and phylogenetic (maximum parsimony, maximum likelihood) and phenetic (neighbor joining) analyses of cytochrome-*b* sequences (Braggio and Bonvicino 2004). A synthetic view of progress made since then has been recently published (by Pessôa et al. 2015a), and restricted species names to populations from type localities and nearby areas. In absence of a comprehensive systematic review for the genus *Thrichomys*, we provisionally adhere to that synthesis, according to which *Thrichomys apereoides* is restricted to a few localities in the state of Minas Gerais. Nevertheless, records potentially attributable to this species exist for localities corresponding to the Cerrado biome in the Brazilian states of Goiás and Tocantins (Bezerra et al. 2013, Gomes et al. 2015). Clearly, a systematic review including karyotyped and sequenced samples from throughout the distribution of all currently recognized species and their type localities is needed to establish species boundaries and distributions.

###### Conservation status.

The red list of the IUCN ver. 3.1 assigned the category “Least Concern” to *Thrichomys apereoides* (see Roach and Naylor 2016b). The species was not included in the official list of threatened species of Brazil (ICMBIO-MMA 2016).

##### Thrichomys inermis

Taxon classificationAnimaliaRodentiaEchimyidae

(Pictet, 1843)

###### Distribution.

*Thrichomys inermis* is endemic to the Caatinga (contra Carmignotto et al. 2012, Paglia et al. 2012), and has been recorded in the Brazilian state of Bahia (Pereira and Geise 2009, Pessôa et al. 2015a). Alleged records of the species for the Brazilian state of Tocantins, from what seems to correspond to the Cerrado biome, reported in abstracts of meeting presentations have permeated through the literature – e.g., cited by Oliveira and Bonvicino (2006), Bonvicino et al. (2008), Pessôa et al. (2015a) – but, to the best of our knowledge, tangible evidence of the species being present in states and biomes other than Bahia and the Caatinga, respectively, is still lacking. The aforementioned abstracts are as follows:

Carvalho AH, Fagundes V (2005) Área de ocorrência de três táxons do gênero *Thrichomys* (Echimyidae, Rodentia) baseados em identificação cariotípica. In: Fagundes V, Costa LP, Leite YLR, Mendes SL (Eds), Livros de resumos, III Congresso Brasileiro de Mastozoologia. Espírito Santo, Aracruz, p. 102.

Carvalho AH, Lopes MOG, Svartman M (2008) Cariótipo de *Thrichomys inermis* (Rodentia, Echimyidae) do Tocantins. XXVII Congresso Brasileiro de Zoologia, Curitiba.

###### Conservation status.

The red list of the IUCN ver. 3.1 assigned the category “Least Concern” to *Thrichomys inermis* (see Bonvicino and Geise 2008b). The species was not included in the official list of threatened species of Brazil (ICMBIO-MMA 2016).

##### Trinomys albispinus

Taxon classificationAnimaliaRodentiaEchimyidae

(I. Geoffroy, 1838)

###### Distribution.

*Trinomys albispinus* is endemic to the Cerrado and the Caatinga (contra Carmignotto et al. 2012, Paglia et al. 2012), and has been recorded in the Brazilian states of Bahia, Minas Gerais, and Sergipe (Souza et al. 2006, Pessôa et al. 2015b).

###### Taxonomy.

The recently published synopsis of the genus *Trinomys* by Pessôa et al. (2015b) followed the views of Reis and Pessôa (1995) and Souza et al. (2006) in treating *Trinomys minor* as a subspecies of *Trinomys albispinus* (i.e., *Trinomys albispinus minor*). Reis and Pessôa (1995) did not discuss the biogeographic context they presumably considered to designate *minor* as a subspecies of *Trinomys albispinus* (then allocated in the genus *Proechimys*) instead of a valid species. Souza et al. (2006) considered *minor* as a subspecies of *Trinomys albispinus* due to the fact that karyotypes that they and Leal-Mesquita et al. (1992) attributed to *albispinus* and *minor*, respectively, shared the same diploid and autosomal fundamental numbers (2n=60, FN_a_=116), morphology of the sex chromosomes, and size of the first and second pairs of autosomes. Pessôa et al. (2015b) textually described a topology presumably resulting from phylogenetic analyses based on cytochrome-*b* sequences by Souza (2011 [an unpublished Ph.D. dissertation]) in which samples attributed to *minor* were nested within a haplogroup formed by samples attributed to *Trinomys albispinus sertonius* (= *Trinomys albispinus albispinus* [sensu Pessôa et al. 2015b and references therein]). We currently lack access to both the sequence data and the analyses that formed the basis of Pessôa et al.’s (2015b) views. Regardless, we argue that *Trinomys minor* and *Trinomys albispinus* represent different, valid species, for the following reasons: (1) the two species occur in a geographic context in which no clear barrier to dispersal separate them (see map in Pessôa et al. 2015b: 1004), and records of both species exists at only 30 km away from each other (see Souza et al. 2006); (2) the two species present well marked morphological differences (Reis and Pessôa 1995, Pessôa and Strauss 1999, Souza et al. 2006), and specimens with intermedium morphological characteristics have not been reported. These aspects strongly suggest that *minor* and *albispinus* do not constitute different subspecies of a single species, but rather that they are valid biological species, able to maintain their morphological differences in close geographic proximity and in absence of barriers to dispersal (i.e., virtually in sympatry) – even if they share the same karyotype and shallow genetic divergences. According to Pessôa et al. (2015b), Iack-Ximenes (2005 [an unpublished Ph.D. dissertation]) also recommends treating *Trinomys minor* as a species rather than as a subspecies of *Trinomys albispinus*. A number of potential causes could explain the yet-to-be-confirmed topology described in the species account by Pessôa et al. (2015b), including incomplete lineage sorting and other more technical aspects of the analyses and/or data (e.g., saturation of sequences, biases in nucleotide composition). Addressing these possibilities is pending from future publication of the sequence data used in those analyses and from future efforts to obtain nuclear sequence data from populations of *Trinomys minor* and *Trinomys albispinus*.

###### Conservation status.

The red list of the IUCN ver. 3.1 assigned the category “Least Concern” to *Trinomys albispinus* (see Bonvicino et al. 2016). The species was not included in the official list of threatened species of Brazil (ICMBIO-MMA 2016).

##### Trinomys minor

Taxon classificationAnimaliaRodentiaEchimyidae

(Reis & Pessôa, 1995)

###### Distribution.

*Trinomys minor* is endemic to the Cerrado and the Caatinga (not Carmignotto et al. 2012, Paglia et al. 2012), and has been recorded in the Brazilian state of Bahia (Pessôa et al. 2015b).

###### Taxonomy.

See the Taxonomy section of *Trinomys albispinus* (above).

###### Conservation status.

The red list of the IUCN ver. 3.1 has not yet attempted to evaluate the extinction risk of *Trinomys minor*, and although the IUCN acknowledged that the Iack-Ximenes’s (2005) unpublished Ph.D. dissertation concluded that *minor* merited species-level recognition, it treated *minor* as a subspecies of *Trinomys albispinus* (see Bonvicino et al. 2016). The species was not included in the official list of threatened species of Brazil (ICMBIO-MMA 2016).

##### Trinomys yonenagae

Taxon classificationAnimaliaRodentiaEchimyidae

(Rocha, 1996)

###### Distribution.

*Trinomys yonenagae* is endemic to the Caatinga, and has been recorded in the Brazilian state of Bahia (Rocha 1995, Pessôa et al. 2015b, Tavares et al. 2016).

###### Conservation status.

The red list of the IUCN ver. 3.1 assigned the category “Endangered” to *Trinomys yonenagae* (see Roach and Naylor 2016a). The species appears in the official list of threatened species of Brazil with the category “Endangered” (ICMBIO-MMA 2016).

## Discussion

*Endemic faunas and taxonomic richness*. Three endemic mammalian faunas can be recognized in our results: one endemic to the Caatinga (ten species that are currently allocated in ten genera; hereafter “Caatinga-only endemics”), other endemic to the Cerrado (22 species that are currently allocated in 18 genera; hereafter “Cerrado-only endemics”), and another endemic to the Caatinga and the Cerrado in combination (i.e., formed by taxa with presence in both biomes; eleven species that are currently allocated in ten genera; hereafter “Caatinga-Cerrado endemics”). Altogether, these faunas encompass 43 species (allocated in 31 genera) that are only found in either the Caatinga, or the Cerrado, or both (Tables 1 and 2). Discrepancies between these figures – as well as the taxonomic identity of the taxa that form their basis – with respect to those from previous syntheses (e.g., Marinho-Filho et al. 2002, Ribeiro et al. 2010, Carmignotto et al. 2012) can be explained by the following factors: (1) some of the currently available data on the taxonomy and distribution of these faunas were not available when previous syntheses were conducted (see under Species Accounts); (2) the geographic scope considered in the present study differ from some of those used in previous studies – e.g., contrary to Carmignotto et al. 2012, we did not regard the Pantanal as part of the Cerrado, as we consider it to be a biome itself, with particular influences from other humid biomes (see Werneck 2011 and references therein); (3) we disregarded information that has not ever been published, but that had permeated into the literature in the form of cited abstracts that merely correspond to presentations in scientific meetings, personal observations, or unpublished data (with the only exception of habitat information for three species of *Lonchophylla* and *Xeronycteris vieirai*; see Table 1). Current and future fieldwork and taxonomic research will undoubtedly alter our current list of endemic species (Table 1). For instance, future research might reveal that the distributions of species currently known only from transitional zones between our focal and neighboring biomes are larger than currently understood, which might render some of these species as endemic to our focal biomes (see section “Species currently only known from transitional areas”, below).

**Table 1.** List of mammal species endemic to the Caatinga, the Cerrado, or both, and their habitat and conservation statuses. Biome: Caatinga (Ca), Cerrado (Ce). Habitat: forest (F), open (O; e.g., campo limpo, cerrado *sensu stricto*; Oliveira and Marquis 2002), Locs: approximate number of localities for which voucher specimens exist according to the literature (see Species Accounts). IUCN: conservation status according to the International Union for the Conservation of Nature (Red List of Threatened Species version 3.1). ICMBio-MMA: conservation status according to the *Instituto Chico Mendes de Conservação da Biodiversidade*-*Ministério do Meio Ambiente* of Brazil (ICMBio-MMA 2016). Categories for conservation statuses: Data Deficient (DD), Low Concern (LC), Vulnerable (VU), Near Threatened (NT), Endangered (EN), Critically Endangered (CR), Extinct (EX); species that have not been evaluated by the IUCN or the ICMBio-MMA are indicated with m-dashes. Differences with respect to previous lists (from the literature) are discussed in the Results section.

Order, Family	Species	Biome	Habitat	Locs.	IUCN	ICMBio-MMA
Chiroptera, Phyllostomidae	*Chiroderma vizottoi*	Ca	F	4	–	–
	*Lonchophylla inexpectata*	Ca	F, O^1^	3	–	–
	*Xeronycteris vieirai*	Ca	O^2^	6	DD	VU
Primates, Pitheciidae	*Callicebus barbarabrownae*	Ca	F	>15	CR	CR
Rodentia, Caviidae	*Galea spixii*	Ca	O	>15	LC	–
	*Kerodon rupestris*	Ca	O	>15	LC	VU
Rodentia, Cricetidae	*Rhipidomys cariri*	Ca	F	10	DD	VU
	*Wiedomys pyrrhorhinos*	Ca	F, O	14	LC	–
Rodentia, Echimyidae	*Thrichomys inermis*	Ca	F, O	12	LC	–
	*Trinomys yonenagae*	Ca	O	2	EN	EN
Didelphimorphia, Didelphidae	Thylamys (Xerodelphis) velutinus	Ce	O	7	NT	VU
Chiroptera, Phyllostomidae	*Lonchophylla bokermanni*	Ce	F, O^1^	4	EN	–
	*Lonchophylla dekeyseri*	Ce	F, O	6	EN	EN
Primates, Cebidae	*Callithrix penicillata*	Ce	F, O	>15	LC	–
Rodentia, Caviidae	*Kerodon acrobata*	Ce	F, O	5	DD	VU
Rodentia, Cricetidae	*Calassomys apicalis*	Ce	O	2	–	–
	*Calomys tocantinsi*	Ce	F, O	10	LC	–
	*Cerradomys marinhus*	Ce	F, O	2	DD	–
	*Euryoryzomys lamia*	Ce	F^3^	3	EN	EN
	*Gyldenstolpia planaltensis*	Ce	O	3	–	–
	*Juscelinomys candango*	Ce	O	1	EX	CR/EX
	*Juscelinomys huanchacae*	Ce	O	4	DD	–
	*Microakodontomys transitorius*	Ce	F, O	2	EN	EN
	*Oecomys cleberi*	Ce	F	4	DD	–
	*Oligoryzomys moojeni*	Ce	F, O	7	DD	–
	*Oligoryzomys rupestris*	Ce	O	3	DD	EN
	*Thalpomys cerradensis*	Ce	O	>15	LC	VU
	*Thalpomys lasiotis*	Ce	O	10	LC	EN
	*Wiedomys cerradensis*	Ce	F, O	3	DD	–
Rodentia, Echimyidae	*Carterodon sulcidens*	Ce	O	12	DD	–
	*Phyllomys brasiliensis*	Ce	F, O	2	EN	EN
	*Thrichomys apereoides*	Ce	O	15	LC	–
Didelphimorphia, Didelphidae	*Cryptonanus agricolai*	Ca, Ce	F, O^4^	10	DD	–
	Thylamys (Xerodelphis) karimii	Ca, Ce	F, O	>15	VU	–
Cingulata, Dasypodidae	*Tolypeutes tricinctus*	Ca, Ce	F, O	>15	VU	EN
Chiroptera, Phyllostomidae	*Micronycteris sanborni*	Ca, Ce	F, O	13	DD	–
Primates, Cebidae	Cebus (Sapajus) libidinosus	Ca, Ce	F, O	>15	LC	–
Carnivora, Canidae	*Lycalopex vetulus*	Ca, Ce	O	>15	LC	VU
Rodentia, Cricetidae	*Calomys expulsus*	Ca, Ce	F, O	>15	LC	–
	*Oligoryzomys stramineus*	Ca, Ce	F, O	>15	LC	–
	*Oxymycterus delator*	Ca, Ce	O	>15	LC	–
Rodentia, Echimyidae	*Trinomys albispinus*	Ca, Ce	F	12	LC	–
	*Trinomys minor*	Ca, Ce	O	3	–	–

1 Unpublished observations suggest that *Lonchophylla inexpectata*, *Lonchophylla bokermanni*, and *Lonchophylla dekeyseri* occur in open and forest habitat types (R. Moratelli *in litt.* for *Lonchophylla inexpectata* and *Lonchophylla bokermanni*; personal observations for *Lonchophylla dekeyseri*).

2 *Xeronycteris vieirai* is associated to outcroppings in open habitats; however, some of this outcroppings are located near forests, and it is likely that the species forages in them (M. Nogueira *in litt*.).

3 Two of the three known localities known for *Euryoryzomys lamia* correspond to transitional areas between gallery forest and open habitats (see Bonvicino et al. 1998).

4 Currently available records for *Cryptonanus agricolai* suggest that it occurs in open habitats as well as in enclaves of moist forests within the Caatinga locally known as *brejos de altitude*.

**Table 2.** Number of genera and species endemic to the Caatinga, the Cerrado, or both, per mammalian order. The column “Caatinga-Cerrado” corresponds to endemic taxa with presence in both biomes, whereas the column “all endemics” corresponds to taxa either endemic to the Caatinga, or to the Cerrado, or endemic to both in combination.

	Caatinga-only		Cerrado-only		Caatinga-Cerrado		All endemics	
	Genera	Species	Genera	Species	Genera	Species	Genera	Species
Didelphimorphia	0	0	0	1	0	2	0	3
Cingulata	0	0	0	0	0	1	0	1
Chiroptera	1	3	0	2	0	1	1	6
Primates	0	1	0	1	0	1	0	3
Carnivora	0	0	0	0	1	1	1	1
Rodentia	0	6	5	18	2	5	7	29
Total	1	10	5	22	3	11	9	43

Species richness was the highest in the Cerrado-only endemics, followed by the Caatinga-Cerrado endemics, and then by the Caatinga-only endemics. The order with highest species richness in all three faunas aforementioned was rodents; all other orders were represented by only one to three species (Tables 1 and 2). Pooling together the three faunas, bats were the second most important order, with six species. These results are congruent with patterns found in earlier studies (e.g., Marinho-Filho et al. 2002, Carmignotto et al. 2012 and references therein). The fact that the Caatinga-Cerrado endemics present an intermedium level of species richness between that of the Caatinga-only and the Cerrado-only endemics suggests the possibility that the Cerrado might have functioned as a source of ancestral populations for the Caatinga. This process might be explained by (1) the chronological order in which the Caatinga and the Cerrado established in the region, with the Cerrado being established substantially earlier than the Caatinga, and (2) by the larger size and higher habitat heterogeneity of the Cerrado (see Werneck 2011 and references therein; Carmignotto et al. 2012). Nevertheless, this process and its potential causal explanations remain as hypotheses to be tested employing other sources of information and considering additional factors, as the possible effect of species extinction masking past patterns of species richness in the focal biomes.

As demonstrated, from the biogeographic point of view, the Caatinga-Cerrado endemics deserve attention as a unit, as is the case for the Caatinga-only and the Cerrado-only endemics (e.g. Carmignotto et al. 2012). The Caatinga-Cerrado endemic fauna is composed of two marsupials (*Cryptonanus agricolai*, *Thylamys karimii*), one xenarthran (*Tolypeutes tricinctus*), one bat (*Micronycteris sanborni*), one primate (*Cebus libidinosus*), one carnivore (*Lycalopex vetulus*), and five rodents (*Calomys expulsus*, *Oligoryzomys stramineus*, *Oxymycterus delator*, *Trinomys albispinus*, *Trinomys minor*). Two genera are currently known to be endemic to the Caatinga-Cerrado unit (the marsupial genus *Cryptonanus* and the rodent genus *Kerodon*). Among Caatinga-Cerrado endemics, only *Trinomys albispinus* seems to be strictly associated to forest habitat, whereas *Lycalopex vetulus*, *Oxymycterus delator*, and *Trinomys minor* seem strictly associated to open habitat. The remaining seven species are likely associated to both open and forest habitats (Table 2; see also Species Accounts). This heterogeneity in habitat association suggests a complex biogeographic history. To dig into this history, it is necessary to revise the phylogenetic information existing for each of these species, and integrate it with that for Caatinga-only and Cerrado-only endemics. Such a task is out of the scope of the present study, which aims to set a baseline, regarding the distribution, taxonomy, and conservation statuses of the focal species, upon which further studies could be built. However, we stress that delimiting these three faunas (Caatinga-only, Cerrado-only, and Caatinga-Cerrado endemics) as discrete study subjects – rather than focusing only on two of them, those of the Caatinga and the Cerrado – and then integrating information about their corresponding biogeographic patterns, might provide novel information regarding the evolutionary origin of the biota of open dry habitats of central and northeastern South America.

*Species currently only known from transitional areas*. A group of species are currently known only from transitional areas between our focal biomes and other adjacent biomes. These species include *Oligoryzomys utiaritensis*, *Rhipidomys ipukensis*, and an undescribed species of *Akodon*, all of which occur in transitional areas between the Brazilian Cerrado and the Amazon (Rocha 2011b, Pardiñas et al. 2015, Weksler and Bonvicino 2015); it is unclear whether they are restricted to those transitional areas, or if they are rather Cerrado, or Amazon, endemics with presence in the contact zones between these two biomes. Similarly, *Calomys cerqueirai* and *Phyllomys brasiliensis* are known from transitional areas between the Cerrado and the Atlantic Forest (Bonvicino et al. 2010, Leite and Loss 2015, Salazar-Bravo 2015); it remains to be investigated whether these species are restricted to those transitional areas, or if they are rather Cerrado, or Atlantic Forest, endemics with presence in the contact zones between these biomes.

**Figure 1.**
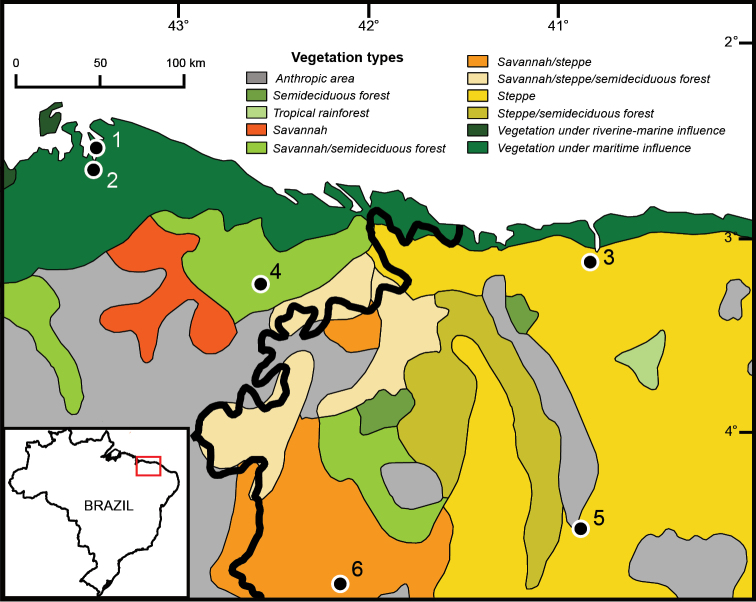
Localities of *Alouatta belzebul ululata* reported in the literature (Viana et al. 2015). Borders of biomes and habitat types as regarded by the *Instituto Brasileiro de Geografia e Estatística* (IBGE 2004a, b). Thick line represents the borders of the Cerrado (left) and the Caatinga (right) according to the IBGE (2004a). Localities: **1** Maranhão, Primeira Cruz (Igarapé das Palmeiras) (2.50°S, 43.33°W; Gregorin 2006) **2** Maranhão, Humberto de Campos (antiga Miritiba) (2.62°S, 43.45°W; Gregorin 2006) **3** Ceará, Granjá (Goiabeira) (3.10°S, 40.83°W; Gregorin 2006) **4** Maranhão, Boa Vista (3.22°S, 42.57°W; Gregorin 2006) **5** Ceará, São Benedito (Bom Jardim) (4.50°S, 40.88°W; Gregorin 2006); Piauí, Campo Maior [4.50°S, 40.88°W; description of locality from Viana et al. (2015); coordinates assigned by us and assessed via Google Earth version 7.1.7.2602]. Geographic coordinates in decimal degrees rounded to the nearest tenth. The original names in Portuguese for the vegetation types as reported by the IBGE (2004b) and their corresponding translation into English are as follows: *área antropizada* (anthropic area); *floresta estacional semidecidual* (semideciduous forest or dry forest); *floresta ombrófila aberta* (tropical rainforest); *savana* (savannah); *savana/floresta estacional* (savannah/semideciduous forest); *savana/savana estépica* (savannah/steppe); *savana/savana estépica/floresta estacional* (savannah/steppe/semideciduous forest); *savana estépica* (steppe); *savana estépica/floresta estacional* (steppe/semideciduous forest); *vegetação com influência fluviomarinha* (vegetation under riverine-marine influence); *vegetação com influência marinha* (vegetation under maritime influence).

*A howler monkey endemic to the Cerrado and the Caatinga*? A case that deserves special discussion is that of a group of populations of howler monkeys from the northern part of the Brazilian states of Ceará, Maranhão, and Piauí. These populations have been regarded as a valid species, *Alouatta ululata* (e.g., Elliot 1912, 1913, Gregorin 2006), or as either a subspecies (Bonvicino et al. 1989) or a junior synonym (Groves 2001, 2005) of *Alouatta belzebul*. The primary argument based on which recognizing “*ululata*” as a valid taxon (either a species or a subspecies of *Alouatta belzebul*) has been advocated is the existence of sexual dimorphism on pelage coloration in this form; however, there is a wide variation in color pelage within *Alouatta belzebul*, and some variation exists even among samples of “*ululata*” (see Gregorin 2006). In fact, in his review of Brazilian species of *Alouatta*, Gregorin (2006) acknowledged to have defined “*Alouatta ululata*” based on its “… *sexual dicromism* [sic] *on pelage, but this character can be an artefate* [sic] *due the small sample*”, and that this taxon needs to be further studied to confirm its validity. One such study was recently published based on analyses of sequence data from the mitochondrial cytochrome-*b* gene (CYTB) and karyotypes (Viana et al. 2015). This study found no karyological differences between *Alouatta belzebul* and “*ululata*”, and its phylogenetic analyses recovered “*ululata*” nested within a haplogroup formed by all analyzed samples of *Alouatta belzebul*. Given that karyological and CYTB data have proven to be taxonomically informative in *Alouatta* (e.g., Bonvicino et al. 1995, Cortés-Ortiz et al. 2003), the results just described suggest that “*ululata*” might just be a junior synonym of *Alouatta belzebul*. The Principal Component Analysis conducted by Gregorin (2006) on the basis of linear measurements taken on skulls and mandibles support this view, as no morphometric discontinuities between “*ululata*” and samples of *Alouatta belzebul* were detected. Nevertheless, considering that only one sample of “*ululata*” was analyzed in the study by Viana et al. (2015), and that future efforts based on more samples and the use of faster evolving parts of the genome – e.g., the mitochondrial control region, nuclear introns, or some flanking regions of ultra conserved elements – could potentially reveal that “*ululata*” represent a linage worth nomenclatural recognition, we conservatively treat “*ululata*” as a subspecies of *Alouatta belzebul* (i.e., *Alouatta belzebul ululata*), a status also considered by Bonvicino et al. (1989). Future efforts to clarify the taxonomic status of populations that have been referred to as “*ululata*” should test for an additional possibility, which is that population that have been referred to as corresponding to “*ululata*” could represent hybrids between *Alouatta belzebul* and *Alouatta caraya* (Garbino *in litt*.). With respect to the distribution of *Alouatta belzebul ululata*, some authors regarded it as endemic to the Cerrado and the Caatinga (Carmignotto et al. 2012), whereas others regarded it as restricted to the Amazonia and the Caatinga (Paglia et al. 2012). Using Geographic Information Systems, we determined that occurrence localities of *Alouatta belzebul ululata* (from Gregorin 2006, Viana et al. 2015) are within the borders of Cerrado and the Caatinga biomes as defined by the *Instituto Brasileiro de Geografia e Estatística* (IBGE 2004b) (Figure 1). Within these biomes, *Alouatta belzebul ululata* inhabits predominantly transitional areas between dry, open vegetation and Amazon rain forest (*floresta pluvial amazônica*; Gregorin 2006). Whereas assessing the real taxonomic status of populations currently attributed to *Alouatta belzebul ululata* remains to be accomplished, conservation efforts should be invested in protecting its endangered populations (see de Oliveira and Kierulff 2008).

*A porcupine endemic to the Caatinga?* Feijó and Langguth (2013) described an alleged new species of porcupine, *Coendou baturitensis*, on the basis of four specimens from the Baturité Range in the Brazilian state of Ceará, in the Caatinga biome. These authors mentioned that the [alleged] new species differs from *Coendou prehensilis* in the color pattern of quills and in having a darker general appearance. In absence of a larger sample and quantitative analyses that take into account the geographic and non-geographic variation within *Coendou prehensilis* and other species of *Coendou*, we are not convinced that *Coendou baturitensis* represents a valid species. Therefore, herein we provisionally treat *Coendou baturitensis* as a junior synonym of *Coendou prehensilis* (see also Voss 2015b).

*Species previously regarded as endemic*. A number of species that were previously considered as endemic to the Caatinga, the Cerrado, or both, are not considered endemic to those geographic units in this study because: (1) our criterion to deem a species as endemic is stricter than that used by other authors (see Methods); or (2) recently published information demonstrate that these species are present in biomes other than the Caatinga and the Cerrado; or because (3) we do not consider them valid species. Due to either of the former two criteria, we excluded the following species from our list of endemics: *Calomys tener*, *Cerradomys langguthi*, *Cerradomys vivoi*, *Cerradomys maracajuensis*, *Cerradomys subflavus*, *Ctenomys brasiliensis*, *Ctenomys nattereri*, *Clyomys laticeps*, *Dasyprocta azarae*, *Guerlinguetus poaiae*, *Kunsia tomentosus*, *Phyllomys blainvillii*, *Pseudoryzomys simplex*, *Rhipidomys macrurus*, *Thrichomys laurentius* (see Cordeiro-Estrela et al. 2006, de la Sancha et al. 2011, Bezerra 2015, Bezerra and Bonvicino 2015b, Bidau 2015, Patton and Emmons 2015, Percequillo 2015, Pessôa et al. 2015a, Salazar-Bravo 2015, de Vivo and Carmignotto 2015, Voss 2015a, Caccavo and Oliveira 2016). Besides the cases of *Alouatta ululata* and *Coendou baturitensis*, which we do not consider valid species (see discussion of cases above), we adhere to recent synopses of mammal genera provided by various authors, and do not recognize the following names as corresponding to valid species: *Guerlinguetus poaiae*, *Urosciurus urucumus*, *Dasyprocta nigriclunis*, and *Rhipidomys cearanus* (see Patton and Emmons 2015, Tribe 2015, de Vivo and Carmignotto 2015).

**Figure 2.**
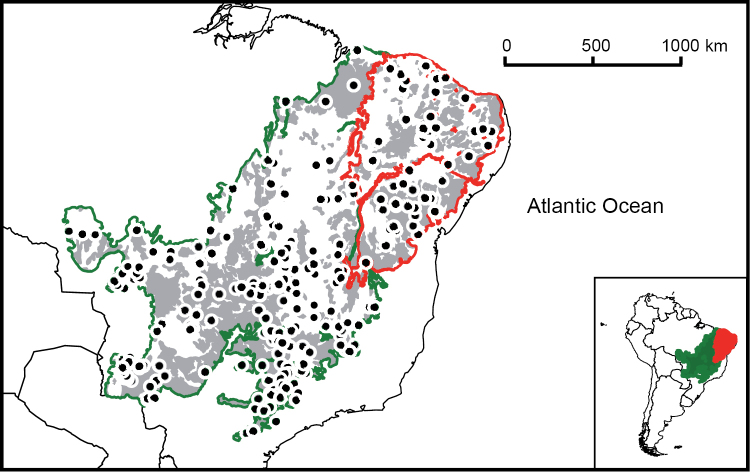
Sampling conducted for mammals and habitat lost due to human activities in the Caatinga and the Cerrado. Dots represent sites where sampling has been conducted according to data from the Global Information Biodiversity Facility (GBIF) and Ribeiro et al. (2010). These data represent a highly conservative proxy of the sampling conducted in these biomes (see Discussion). Areas in grey represent areas where habitat loss has occurred due to human activities. Borders of the Caatinga (red) and the Cerrado (green) are restricted to Brazil (i.e., does not include the small portions of the Cerrado in Bolivia and Paraguay). The plotted data for habitat loss and biome borders were obtained from the *Instituto Brasileiro de Geografia e Estatística*.

*Conservation*. A substantial portion of the endemic mammalian faunas of the Caatinga and the Cerrado faces high risk of extinction; however, this fact has been dangerously overlooked. Considering the information published by the International Union for Conservation of Nature (IUCN; see Methods and Species Accounts), the great majority of members of the endemic faunas of the Caatinga and the Cerrado are either of low concern, lack data for assessing their extinction risk, or have not been considered yet by the IUCN (Table 3). According to IUCN data, these three categories (Low Concern, Data Deficient, not evaluated) conform 80% of the Caatinga-only endemics (8 out of 10 species), 68% of the Cerrado-only endemics (15 out of 22 species), 82% of the Caatinga-Cerrado endemics (9 out of 11 species), and 74% of all endemics (32 out of 43 species). We argue that this situation does not reflect the reality of these faunas. Our reasons are as follows:

For mammals, the Caatinga and the Cerrado have been relatively well sampled; yet several of their endemic species are known only from a handful of localities. Since the second half of the 20^th^ century, surveys of mammals in both the Caatinga and the Cerrado covered numerous sites (Mares et al. 1989, Marinho-Filho and Veríssimo 1997, Marinho-Filho et al. 1997, Bonvicino and Bezerra 2003, Bonvicino et al. 2005, 2012, Bezerra et al. 2007, 2009, 2010, 2014, Astúa and Guerra 2008, Oliveira et al. 2008, Bocchiglieri et al. 2010, Bruna et al. 2010, Silva et al. 2015; see also Marinho-Filho et al. 2002, Carmignotto et al. 2012 and references therein; see also citations under Species Accounts). Although biodiversity inventories in the Caatinga and the Cerrado are far from being complete, both biomes are relatively well sampled when compared to other biomes (e.g., the Amazon). A map (Figure 2) showing localities where sampling of mammals have taken place in the Caatinga and the Cerrado, based on data from both the Global Biodiversity Information Facility (GBIF) and the contribution by Ribeiro et al. (2010), documents that our focal biomes have been relatively well sampled. The sampling shown on this map is a quite conservative proxy of the degree of sampling conducted in these biomes, as it lacks data from Brazilian and several North American collections that are not yet available through GBIF or that were not included in the publication by Ribeiro et al. (2010) – this latter study used data from some of the Brazilian collections and focused on endemic and threatened species of the Cerrado, lacking data for species that did not meet either of these conditions. Given the sampling effort achieved in the last decades, we argue that most, if not all, of the endemic species of the Caatinga and the Cerrado to which the IUCN assigned the category “Data Deficient” are rather species with recognizable risks of extinction. The category “Data Deficient” implies the possibility that future efforts (i.e., museum work, fieldwork, or both) might demonstrate that these species are sufficiently common to be of Low Concern; however, the sampling already conducted should have yielded enough distributional records if the species did not have very low population sizes, extremely restricted distributions, or both. The application of the IUCN criteria for assigning conservation statuses globally are clearly inadequate when applied to species that are known from few records and are endemic to regions that have been well sampled.

Natural habitat loss has been pervasive in the Caatinga and the Cerrado. Due to agriculture or transformation of rural areas into urban areas, both of these biomes have lost enormous amounts of their natural habitats (Klink and Machado 2005, Leal et al. 2005, Beuchle et al. 2015). In addition to agriculture, the Caatinga suffer of high degrees of habitat transformation due to human occupation, with replacement of gallery and dry forests by open vegetation via charcoal production, timber and cattle ranching (Sampaio 1995, Leal et al. 2005, Miles et al. 2006, Pennington et al. 2006, Werneck 2011); and in the Cerrado invasive African grasses and uncontrolled fire also contribute to the elimination of natural habitats (Klink and Machado 2005). Using data from the *Instituto Brasileiro de Geografia e Estatística* (http://www.ibge.gov.br), we generated a map that shows extensive areas of the Cerrado and the Caatinga that have suffered of habitat transformation due to human activities (Figure 2). The extent and accelerated trend of habitat loss in both biomes make it even more likely that species for which only a handful of localities are known are facing high risks of extinction, therefore deserving conservation categories that indicate such a situation (rather than the category “Data Deficient”).

The Red List published by the IUCN is highly influential in conservation planning initiatives; hence, a review and improvement of the IUCN criteria, and of how are they applied, based on the issues we just described, represents an imperative task for the conservation of these and other endemic faunas. Sensible assessments of their conservation statuses should reflect realities of species, which is not currently the case of several of our focal species, including eleven rodents (*Calassomys apicalis*, *Cerradomys marinhus*, *Gyldenstolpia planaltensis*, *Juscelinomys huanchacae*, *Kerodon acrobata*, *Oligoryzomys moojeni*, *Oligoryzomys rupestris*, *Thalpomys cerradensis*, *Trinomys minor*, *Trinomys yonenagae*, *Wiedomys cerradensis*) and two bats (*Xeronycteris vieirai* and *Lonchophylla inexpectata*). Both bat species have been recently described; hence, because future reexamination of museum specimens might reveal that these species are more common than currently thought, the category “Data Deficient”, which has been assigned to them, might be justifiable. All other cases are undoubtedly facing certain recognizable risk of extinction.

When compared with the IUCN assessment, the Brazilian national assessment of the conservation statuses yielded categories that seem more congruent with the high risks of extinction these species face. Nevertheless, many of the species evaluated by the IUCN have not been considered in this national assessment, indicating the need for a larger and more expedite effort by Brazil’s *Ministério do Meio Ambiente* (ICMBIO-MMA 2016) to assure the conservation of the unique fauna Brazil harbors in the Caatinga and the Cerrado. This is an urgent task because many of these endemic species are associated to habitats that are experiencing fast transformation into areas for agriculture, at an unbearable cost for biodiversity.

**Table 3.** Representation of species endemic to the Caatinga, the Cerrado, or both, in each category of conservation status. The column “Caatinga-Cerrado” corresponds to taxa with presence in both biomes, whereas the column “all endemics” corresponds to taxa either endemic to the Caatinga, or to the Cerrado, or endemic to both in combination. s: number of species; IUCN: conservation status according to the International Union for the Conservation of Nature (Red List of Threatened Species version 3.1); ICMBio-MMA: conservation status according to the *Instituto Chico Mendes de Conservação da Biodiversidade-Ministério do Meio Ambiente* of Brazil (ICMBio-MMA 2016). Categories for conservation statuses: Data Deficient, Low Concern, Vulnerable, Near Threatened, Endangered, Critically Endangered, and Extinct. See Methods for descriptions of each of these categories.

Category	Caatinga-only				Cerrado-only				Caatinga-Cerrado				All endemics			
	IUCN		ICMBio-MMA		IUCN		ICMBio-MMA		IUCN		ICMBio-MMA		IUCN		ICMBio-MMA	
	s	%	s	%	s	%	s	%	s	%	s	%	s	%	s	%
Not evaluated	2	20	5	50	2	9	12	55	1	9	9	82	5	12	26	60
Data Deficient	2	20	0	0	8	36	0	0	2	18	0	0	12	28	0	0
Low Concern	4	40	0	0	5	23	0	0	6	55	0	0	15	35	0	0
Near Threatened	0	0	0	0	1	5	0	0	0	0	0	0	1	2	0	0
Vulnerable	0	0	3	30	0	0	3	14	2	18	1	9	2	5	7	16
Endangered	1	10	1	10	5	23	6	27	0	0	1	9	6	14	8	19
Critically Endangered^1^	1	10	1	10	0	0	1	5	0	0	0	0	1	2	2	4
Extinct	0	0	0	0	1	5	0	0	0	0	0	0	1	2	0	0
Total number of species	10		10		22		22		11		11		43		43	

1 ICMBio-MMA regards some species as Critically Endangered or Extinct; for simplicity, in this table one species (*Juscelinomys candango*; see Table 1) with that combination of two categories was treated as Critically Endangered.

## Supplementary Material

XML Treatment for Cryptonanus agricolai

XML Treatment for Thylamys (Xerodelphis) karimii

XML Treatment for Thylamys (Xerodelphis) velutinus

XML Treatment for Tolypeutes tricinctus

XML Treatment for Lonchophylla bokermanni

XML Treatment for Lonchophylla dekeyseri

XML Treatment for Lonchophylla inexpectata

XML Treatment for Xeronycteris vieirai

XML Treatment for Micronycteris sanborni

XML Treatment for Chiroderma vizottoi

XML Treatment for Callithrix penicillata

XML Treatment for Cebus (Sapajus) libidinosus

XML Treatment for Callicebus (Callicebus) barbarabrownae

XML Treatment for Lycalopex vetulus

XML Treatment for Galea spixii

XML Treatment for Kerodon acrobata

XML Treatment for Kerodon rupestris

XML Treatment for Gyldenstolpia planaltensis

XML Treatment for Juscelinomys candango

XML Treatment for Juscelinomys huanchacae

XML Treatment for Oxymycterus delator

XML Treatment for Thalpomys cerradensis

XML Treatment for Thalpomys lasiotis

XML Treatment for Cerradomys marinhus

XML Treatment for Euryoryzomys lamia

XML Treatment for Microakodontomys transitorius

XML Treatment for Oecomys cleberi

XML Treatment for Oligoryzomys moojeni

XML Treatment for Oligoryzomys rupestris

XML Treatment for Oligoryzomys stramineus

XML Treatment for Calassomys apicalis

XML Treatment for Calomys expulsus

XML Treatment for Calomys tocantinsi

XML Treatment for Rhipidomys cariri

XML Treatment for Wiedomys cerradensis

XML Treatment for Wiedomys pyrrhorhinos

XML Treatment for Carterodon sulcidens

XML Treatment for Phyllomys brasiliensis

XML Treatment for Thrichomys apereoides

XML Treatment for Thrichomys inermis

XML Treatment for Trinomys albispinus

XML Treatment for Trinomys minor

XML Treatment for Trinomys yonenagae

## References

[B1] Ab’Sáber AN (1974) O domínio morfoclimático semi-árido das caatingas Brasileiras. Geomorfologia 53: 1–19.

[B2] Aguiar L (2016) *Lonchophylla bokermanni* The IUCN Red List of Threatened Species 2016: e.T12263A22038287. https://doi.org/10.2305/IUCN.UK.2016-3.RLTS.T12263A22038287.en

[B3] Aguiar L Bernard E (2016) *Lonchophylla dekeyseri* The IUCN Red List of Threatened Species 2016: e.T12264A22038149. http://www.iucnredlist.org/details/12264/0 [Downloaded 15 October 2016]

[B4] Aguiar LMS Bernard E Machado RB (2014) Habitat use and movements of *Glossophaga soricina* and *Lonchophylla dekeyseri* (Chiroptera: Phyllostomidae) in a Neotropical savannah. Zoologia 31(3): 223–229. https://doi.org/10.1590/S1984-46702014000300003

[B5] Albuquerque UP de Lima Araújo E El-Deir ACA Lima ALA Souto A Bezerra BM Ferraz EMN Freire EMX Sampaio EVSB Las-Casas FMG Moura GJB Pereira GA Melo JG Ramos MA Rodal MJN Schiel N Lyra-Neves RM Alves RRN Azevedo-Júnior SM Júnior WRT Severi W (2012) Caatinga revisited: ecology and conservation of an important seasonal dry forest. The Scientific World Journal 2012: 205182. https://doi.org/10.1100/2012/205182 10.1100/2012/205182 PMC3415163 22919296

[B6] Almeida B Novaes RLM Aguieiras M Souza RF Esbérard CEL Geise L (2016) Karyotype of three *Lonchophylla* species (Chiroptera, Phyllostomidae) from Southeastern Brazil. Comparative Cytogenetics 10(1): 109–115. https://doi.org/10.3897/CompCytogen.v10i1.6646 10.3897/CompCytogen.v10i1.6646 PMC4856929 27186341

[B7] Almeida FC Bonvicino CR Cordeiro-Estrela P (2007) Phylogeny and temporal diversification of *Calomys* (Rodentia, Sigmodontinae): implications for the biogeography of an endemic genus of the open/dry biomes of South America. Molecular Phylogenetics and Evolution 42: 449–466. https://doi.org/10.1016/j.ympev.2006.07.00 10.1016/j.ympev.2006.07.005 16971145

[B8] Almeida B Novaes RLM Aguieiras M Souza RF Esbérard CEL Geise L (2016) Karyotype of three *Lonchophylla* species (Chiroptera, Phyllostomidae) from Southeastern Brazil. Comparative Cytogenetics 10(1): 109–115. https://doi.org/10.3897/CompCytogen.v10i1.6646 10.3897/CompCytogen.v10i1.6646 PMC4856929 27186341

[B9] Andrade AFB Bonvicino CR Briani DC Kasahara S (2004) Karyologic diversification and phylogenetic relationships of the genus *Thalpomys* (Rodentia, Sigmodontinae). Acta Theriologica 49: 181–190.

[B10] Andrade-Lima D (1981) The Caatingas Dominium. Revista Brasileira de Botânica 4:149–163.

[B11] Astúa D Guerra DQ (2008) Caatinga bats from Mammal Collection of the Universidade Federal de Pernambuco. Chiroptera Neotropical 14: 326–338.

[B12] Beuchle R Grecchi RC Shimabukuro YE Seliger R Eva HD Sano E Achard F (2015) Land cover changes in the Brazilian Cerrado and Caatinga biomes from 1990 to 2010 based on a systematic remote sensing sampling approach. Applied Geography 58: 116–127. http://dx.doi.org/10.1016/j.apgeog.2015.01.017

[B13] Bezerra AMR (2008) Revisão taxonômica do gênero *Galea* Meyen, 1832 (Rodentia, Caviidae, Caviinae). Tese do Doutorado [Ph.D. dissertation], Universidade de Brasília, Distrito Federal, Brasília.

[B14] Bezerra AMR (2015) Genus *Kunsia* Hershkovitz, 1966. In: Patton JL, Pardiñas UFJ, D’Elía G (Eds) Mammals of South America. Volume 2. Rodents. The University of Chicago Press, Chicago, 228–231.

[B15] Bezerra AMR Bonvicino CR (2015) Genus *Carterodon* Waterhouse, 1848. In: Patton JL, Pardiñas UFJ, D’Elía G (Eds) Mammals of South America. Volume 2. Rodents. The University of Chicago Press, Chicago, 933–935.

[B16] Bezerra AMR Bonvicino CR (2015b) Genus *Clyomys* Thomas, 1916. In: Patton JL, Pardiñas UFJ, D’Elía G (Eds) Mammals of South America. Volume 2. Rodents. The University of Chicago Press, Chicago, 935–937.

[B17] Bezerra AMR Bonvicino CR Menezes AAN Marinho-Filho J (2010) Endemic climbing cavy *Kerodon acrobata* (Rodentia: Caviidae: Hydrochoerinae) from dry forest patches in the Cerrado domain: new data on distribution, natural history, and morphology. Zootaxa 2724: 29–36.

[B18] Bezerra AMR Carmignotto AP Nunes AP Rodrigues FHG (2007) New data on the distribution, natural history and morphology of *Kunsia tomentosus* (Lichtenstein, 1830) (Rodentia: Cricetidae: Sigmodontinae). Zootaxa 1505: 1–18.

[B19] Bezerra AMR Carmignotto AP Rodrigues FHG (2009) Small non-volant mammals of an ecotone region between the Cerrado hotspot and the Amazonian rainforest, with comments on their taxonomy and distribution. Zoological Studies 48: 861–874.

[B20] Bezerra AMR Lazar A Bonvicino CR Cunha AS (2014) Subsidies for a poorly known endemic semiarid biome of Brazil: non-volant mammals of an eastern region of Caatinga. Zoological Studies 53: 16. https://doi.org/10.1186/1810-522x-53-16

[B21] Bezerra AMR Lazar A Bonvicino CR Marinho-Filho J (2013) *Wiedomys cerradensis* Gonçalves, Almeida and Bonvicino, 2005 (Mammalia: Rodentia: Cricetidae) in Tocantins and Goiás states, central-northern Brazil. Check List 9: 680–683. http://dx.doi.org/10.15560/9.3.680

[B22] Bezerra AMR Marinho-Filho J Carmignotto AP (2011) A review of the distribution, morphology, and habit of the Owl’s spiny rat *Carterodon sulcidens* (Lund, 1841) (Rodentia: Echimyidae). Zoological Studies 50: 566–576.

[B23] Bidau CJ (2015) Family Ctenomyidae Lesson, 1842. In: Patton JL, Pardiñas UFJ, D’Elía G (Eds) Mammals of South America (Vol. 2) – Rodents. The University of Chicago Press, Chicago, 818–877.

[B24] Bocchiglieri A Mendonça AF Henriques RPB (2010) Composition and diversity of medium and large size mammals in the Cerrado of central Brazil. Biota Neotropica 10(3): 169–176. https://doi.org/10.1590/S1676-06032010000300019

[B25] Bonvicino CR (2003) A new species of *Oryzomys* (Rodentia, Sigmodontinae) of the subflavus group from the cerrado of central Brazil. Mammalian Biology 68: 78–90. https://doi.org/10.1078/1616-5047-00066

[B26] Bonvicino CR (2015) Tribe Wiedomyini Reig, 1980. In: Patton JL, Pardiñas UFJ, D’Elía G (Eds) Mammals of South America (Vol. 2) – Rodents. The University of Chicago Press, Chicago, 682–685.

[B27] Bonvicino CR Almeida FC (2000) Karyotype, morphology and taxonomic status of *Calomys expulsus* (Rodentia: Sigmodontinae). Mammalia 64: 339–351. https://doi.org/10.1515/mamm.2000.64.3.339

[B28] Bonvicino CR Bezerra AMR (2003) Use of regurgitated pellets of Barn Owl (*Tyto alba*) for inventorying small mammals in the cerrado of central Brazil. Studies on Neotropical Fauna and Environment 38: 1–5. https://doi.org/10.1076/snfe.38.1.1.14030

[B29] Bonvicino CR Catzeflis F Patton JL Percequillo A Weksler M (2016) *Trinomys albispinus* The IUCN Red List of Threatened Species 2016: e.T18272A22211739. http://www.iucnredlist.org/details/18272/0 [Downloaded on 15 October 2016]

[B30] Bonvicino CR Geise L (2008a) *Calomys expulsus* The IUCN Red List of Threatened Species 2008: e.T136689A4328022. https://doi.org/10.2305/IUCN.UK.2008.RLTS.T136689A4328022.en [Downloaded on 15 October 2016]

[B31] Bonvicino CR Geise L (2008b) *Thrichomys inermis* The IUCN Red List of Threatened Species 2008: e.T136355A4279753. https://doi.org/10.2305/IUCN.UK.2008.RLTS.T136355A4279753.en [Downloaded 15 October 2016]

[B32] Bonvicino CR Fernandes MEB Seuánez HN (1995) Morphological analysis of *Alouatta seniculus* species group (Primates, Cebidae). A comparison with biochemical and karyological data. Human Evolution 10: 169–176. https://doi.org/10.1007/BF02437539

[B33] Bonvicino CR Langguth A Mittermeier RA (1989) A study of pelage color and geography distribution in Alouatta belzebul (Primates, Cebidae). Revista Nordestina de Biologia 6(2): 139–148.

[B34] Bonvicino CR Lima JFS Almeida FC (2003) A new species of *Calomys* Water house (Rodentia, Sigmodontinae) from the Cerrado of central Brazil. Revista Brasileira de Zoologia 20: 301–307. https://doi.org/10.1590/S0101-81752003000200021

[B35] Bonvicino CR Lazar A Corrêa MMO Weksler M Paula AC Bezerra AMR (2014) Conservation units in the core area of the Cerrado domain: an overview on the small nonvolant mammals (Rodentia and Didelphimorphia). Heringeriana 8: 202–220.

[B36] Bonvicino CR Lemos B Weksler M (2005) Small mammals of Chapada Dos Veadeiros National Park (Cerrado of central Brazil): ecologic, karyologic, and taxonomic considerations. Brazilian Journal of Biology 65: 395–406. https://doi.org/10.1590/S1519-69842005000300004 10.1590/s1519-69842005000300004 16341417

[B37] Bonvicino CR Lindbergh SM Faria MB Bezerra AMR (2012) The eastern boundary of the Brazilian Cerrado: a hotspot region. Zoological Studies 51: 1207–1218.

[B38] Bonvicino CR Marinho-Filho J (2008a) *Calomys tocantinsi* The IUCN Red List of Threatened Species 2008: e.T136679A4326546. https://doi.org/10.2305/IUCN.UK.2008.RLTS.T136679A4326546.en [Downloaded on 15 October 2016]

[B39] Bonvicino CR Marinho-Filho J (2008b) *Wiedomys cerradensis* The IUCN Red List of Threatened Species 2008: e.T136745A4334715. https://doi.org/10.2305/IUCN.UK.2008.RLTS.T136745A4334715.en [Downloaded on 15 October 2016]

[B40] Bonvicino CR Oliveira JA D’Andrea PS (2008) Guia dos roedores do Brasil, com chaves para gêneros baseadas em caracteres externos. Centro Pan-Americano de Febre Aftosa, OPAS/OMS, Rio de Janeiro.

[B41] Bonvicino CR Oliveira JA Gentile R (2010) A new species of *Calomys* (Rodentia: Sigmodontinae) from eastern Brazil. Zootaxa 2336: 19–25.

[B42] Bonvicino CR Otazu I D’Andrea PS (2002) Karyologic evidences of diversification of the genus *Thrichomys* (Rodentia, Echimyidae). Cytogenetics and Genome Research 97: 200–204. https://doi.org/10.1159/000066613 10.1159/000066613 12438714

[B43] Bonvicino C Percequillo A (2008) *Cerradomys marinhus* The IUCN Red List of Threatened Species 2008: e.T136511A4302604. https://doi.org/10.2305/IUCN.UK.2008.RLTS.T136511A4302604.en [Downloaded 15 October 2016]

[B44] Bonvicino CR Weksler M (1998) A new species of *Oligoryzomys* (Rodentia, Sigmodontinae) from central Brazil. Zeitschrift für Säugetierkunde 63: 90–103.

[B45] Braggio E Bonvicino CR (2004) Molecular divergence in the genus *Thrichomys* (Rodentia: Echimyidae). Journal of Mammalogy 85: 316–320. https://doi.org/10.1644/1545-1542(2004)085<0316:MDITGT>2.0.CO;2

[B46] Brasil RFd (2012) O novo código florestal. Lei 12.651, de 25 de maio de 2012. http://www.sinj.df.gov.br

[B47] Bruna EM Guimarães JF Lopes CT Duarte P Gomes ACL Belentani SCS Pacheco R Facure KG Lemos FG Vasconcelos HL (2010) Mammalia, Estação Ecológica do Panga, a Cerrado protected area in Minas Gerais state, Brazil. Check List 6: 668–675. https://doi.org/10.15560/6.4.668

[B48] Bubadué JM Cáceres N Carvalho RS Meloro C (2015) Ecogeographical variation in skull shape of South-American canids: abiotic or biotic processes? Evolutionary Biology. https://doi.org/10.1007/s11692-015-9362-3 10.1007/s11692-015-9362-3 PMC4860408 27217595

[B49] Byrne H Rylands AB Carneiro JC Lynch-Alfaro JW Bertuol F da Silva MNF Messias M Groves CP Mittermeier RA Farias I Hrbek T Schneider H Sampaio I Boubli JP (2016) Phylogenetic relationships of the New World titi monkeys (*Callicebus*): first appraisal of taxonomy based on molecular evidence. Frontiers in Zoology 13:10. https://doi.org/10.1186/s12983-016-0142-4 10.1186/s12983-016-0142-4 PMC4774130 26937245

[B50] Caccavo A Oliveira JA (2016) Detecting morphological limits between parapatric species: cranial variation in *Cerradomys* (Cricetidae: Sigmodontinae) from northeastern Brazil. Journal of Mammalogy [Early View] https://doi.org/10.1093/jmammal/gyw124

[B51] Canale GR Guidorizzi CE Kierulff MCM Gatto CAFR (2009) First record of tool use by wild populations of the yellow-breasted capuchin monkey (*Cebus xanthosternos*) and new records for the bearded capuchin (*Cebus libidinosus*). American Journal of Primatology 71: 1–7. https://doi.org/10.1002/ajp.20648 10.1002/ajp.20648 19206141

[B52] Carleton MD Musser GG (2015) Genus *Oecomys* Thomas, 1906. In: Patton JL, Pardiñas UFJ, D’Elía G (Eds) Mammals of South America (Vol. 2) – Rodents. The University of Chicago Press, Chicago, 393–417.

[B53] Carmignotto AP Aires CC (2011) Mamíferos não voadores (Mammalia) da Estação Ecológica Serra Geral do Tocantins. Biota Neotropica 11: 313–328. https://doi.org/10.1590/S1676-06032011000100029

[B54] Carmignotto AP Astúa D (2016) *Thylamys velutinus* The IUCN Red List of Threatened Species 2016: e.T40520A22172367. http://www.iucnredlist.org/details/40520/0 [Downloaded 15 October 2016]

[B55] Carmignotto AP Monfort T (2006) Taxonomy and distribution of the Brazilian species of *Thylamys* (Didelphimorphia: Didelphidae). Mammalia 70: 126–144. https://doi.org/10.1515/mamm.70.1-2.126

[B56] Carmignotto AP de Vivo M Langguth A (2012) Mammals of the Cerrado and Caatinga: distribution patterns of the tropical open biomes of Central South América. In: Patterson BD, Costa LP (Eds) Bones, clones, and biomes: the history and geography of Recent Neotropical mammals. University of Chicago Press, Chicago, Illinois, 203–229. https://doi.org/10.7208/chicago/9780226649214.003.0010

[B57] Carmignotto AP Astua D Cáceres N (2016a) *Cryptonanus agricolai* The IUCN Red List of Threatened Species 2016: e.T136545A22177735. http://www.iucnredlist.org/details/136545/0 [Downloaded 15 October 2016]

[B58] Carmignotto AP Costa LP Astúa D (2016b) *Thylamys karimii* The IUCN Red List of Threatened Species 2016: e.T136653A22172758. http://www.iucnredlist.org/details/136653/0 [Downloaded 15 October 2016]

[B59] Carvalho BA Oliveira LFB Mattevi MS (2009) Phylogeny of *Thylamys* (Didelphimorphia, Didelphidae) species, with special reference to *Thylamys karimii* Iheringia, Série Zoologia 99: 419–425. https://doi.org/10.1590/S0073-47212009000400012

[B60] Carvalho BDA Oliveira LFB Langguth A Freygang CC Ferraz RS Mattevi MS (2011) Phylogenetic relationships and phylogeographic patterns in *Monodelphis* (Didelphimorphia: Didelphidae). Journal of Mammalogy 92: 121–133. https://doi.org/10.1644/10-MAMM-A-075.1

[B61] Casado F Bonvicino CR Nagle C Comas B Manzur TD Lahoz MM Seuánez HN (2010) Mitochondrial divergence between 2 populations of the hooded capuchin, Cebus (Sapajus) cay (Platyrrhini, Primates). Journal of Heredity 101: 261–269. https://doi.org/10.1093/jhered/esp119 10.1093/jhered/esp119 20056682

[B62] Catzeflis F Patton J Percequillo A Weksler M (2016a) *Galea spixii* The IUCN Red List of Threatened Species 2016: e.T8825A22189453 http://www.iucnredlist.org/details/8825/0 [Downloaded on 15 October 2016]

[B63] Catzeflis F Patton J Percequillo A Weksler M (2016b) *Kerodon rupestris* The IUCN Red List of Threatened Species 2016: e.T10988A22190269 http://www.iucnredlist.org/details/10988/0 [Downloaded on 15 October 2016]

[B64] Coelho DC Marinho-Filho J (2002) Diet and activity of *Lonchophylla dekeyseri* (Chiroptera, Phyllostomidae) in the Federal District, Brazil. Mammalia 66: 319–330. https://doi.org/10.1515/mamm.2002.66.3.319

[B65] Coimbra-Filho AF Mittermeier RA Rylands AB Mendes SL Kierulff MCM Pinto LPS (2006) The taxonomic status of wied’s black-tufted-ear marmoset, *Callithrix kuhlii* (Callitrichidae, Primates). Primate Conservation 1–24. https://doi.org/10.1896/0898-6207.21.1.1

[B66] Colombi VH Fagundes V (2015) First record of *Calomys cerqueirai* (Rodentia: Phyllotini) in Espírito Santo (Brazil) with description of the 2n = 36, FN_A_ = 66 karyotype. Mammalia 79: 479–486. https://doi.org/10.1515/mammalia-2014-0076

[B67] Cordeiro-Estrela P Baylac M Denys C Marinho-Filho J (2006) Interspecific patterns of skull variation between sympatric Brazilian vesper mice: geometric morphometrics assessment. Journal of Mammalogy 87: 1270–1279. https://doi.org/10.1644/05-MAMM-A-293R3.1

[B68] Cortés-Ortiz L Bermingham E Rico C Rodríguez-Luna E Sampaio I Ruiz-García M (2003) Molecular systematics and biogeography of the Neotropical monkey genus *Alouatta* Molecular Phylogenetics and Evolution 26: 64–81. https://doi.org/10.1016/S1055-7903(02)00308-1 10.1016/s1055-7903(02)00308-1 12470939

[B69] Corsini CF Moura ACA (2014) Census of the Blond Titi Monkey *Callicebus barbarabrownae* (Pitheciidae) in the semi-deciduous Atlantic Forest of Chapada Diamantina, Brazil. Neotropical Primates 21: 177–182. https://doi.org/10.1896/044.021.0203

[B70] Costa CHN Courtenay O (2003) A new record of the hoary fox *Pseudalopex vetulus* in north Brazil. Mammalia 67: 593–594. https://doi.org/10.1515/mamm-2003-0416

[B71] Costa LPA Bonvicino CR Weksler M Paglia A (2008) *Oecomys cleberi* The IUCN Red List of Threatened Species 2008: e.T15132A4495985. https://doi.org/10.2305/IUCN.UK.2008.RLTS.T15132A4495985.en [Downloaded 15 October 2016]

[B72] Courtenay O Macdonald DW Gilligham SG Almeida G Dias R (2006) First observations on South America’s largely insectivorous canid: the hoary fox (*Pseudalopex vetulus*). Journal of Zoology (London) 268:45–54. https://doi.org/10.1111/j.1469-7998.2005.00021.x

[B73] Coutinho LM (2006) O Conceito de Bioma. Acta Botánica Brasileira 20: 13–23. https://doi.org/10.1590/S0102-33062006000100002

[B74] Cunha NL Fischer E Carvalho LFAC Santos CF (2009) Bats of Buraco das Araras reserve, southwestern Brazil. Biota Neotropica 9: 189–195. https://doi.org/10.1590/S1676-06032009000400019

[B75] D’Elía G Mora I Myers P Owen RD (2008) New and noteworthy records of Rodentia (Erethizontidae, Sciuridae, and Cricetidae) from Paraguay. Zootaxa 1784: 39–57.

[B76] Dalponte JC (2009) *Lycalopex vetulus* (Carnivora: Canidae). Mammalian Species 847: 1–7. https://doi.org/10.1644/847.1

[B77] Dalponte J Courtenay O (2008) *Pseudalopex vetulus* The IUCN Red List of Threatened Species 2008: e.T6926A12815527. https://doi.org/10.2305/IUCN.UK.2008.RLTS.T6926A12815527.en [Downloaded 15 October 2016]

[B78] Dias D Esberard CEL Moratelli R (2013) A new species of *Lonchophylla* (Chiroptera, Phyllostomidae) from the Atlantic Forest of southeastern Brazil, with comments on *L. bokermanni* Zootaxa 3722(3): 347–360. https://doi.org/10.11646/zootaxa.3722.3.4 10.11646/zootaxa.3722.3.4 26171531

[B79] Díaz-Nieto JF Jansa SA Voss RS (2016) DNA sequencing reveals unexpected Recent diversity and an ancient dichotomy in the American marsupial genus *Marmosops* (Didelphidae: Thylamyini). Zoological Journal of the Linnean Society 176: 914–940. https://doi.org/10.1111/zoj.12343

[B80] Di-Nizo CB Ventura K Ferguson-Smith MA O’Brien PCM Yonenaga-Yassuda Y Silva MJdJ (2015) Comparative chromosome painting in six species of *Oligoryzomys* (Rodentia, Sigmodontinae) and the karyotype evolution of the genus. PLoS ONE 10(2): e0117579. https://doi.org/10.1371/journal.pone.0117579 10.1371/journal.pone.0117579 PMC4320059 25658766

[B81] Dunnum JL (2015) Family Caviidae G. Fischer, 1817. In: Patton JL, Pardiñas UFJ, D’Elía G (Eds) Mammals of South America (Vol. 2) – Rodents. The University of Chicago Press. Chicago, Illinois, 690–725.

[B82] Dunnum J Emmons L Vargas J Bernal N (2008) *Juscelinomys huanchacae* The IUCN Red List of Threatened Species 2008: e.T136353A4279423. https://doi.org/10.2305/IUCN.UK.2008.RLTS.T136353A4279423.en [Downloaded 15 October 2016]

[B83] Dunnum JL Salazar-Bravo J (2010) Phylogeny, evolution, and systematics of the *Galeamusteloides* complex (Rodentia: Caviidae). Journal of Mammalogy 91: 243–259. https://doi.org/10.1644/08-MAMM-A-214R1.1

[B84] Ellerman JR (1941) The families and genera of living rodents. British Museum (Natural History), London 1: 1–689. http://dx.doi.org/10.5962/bhl.title.8332

[B85] Elliot DG (1912) Descriptions of new Species of Monkeys of the genera *Galago*, *Cebus*, *Alouatta*, and *Cercopithecus*. Annals and Magazine of Natural History, London, 8: 77–83.

[B86] Elliot DG (1913) A review of the primates. Bulletin of the American Museum Natural History vol. 1, 317 pp.

[B87] Emmons LH (1999) Two new species of *Juscelinomys* (Rodentia: Muridae) from Bolivia. American Museum Novitates 3280: 1–15. http://hdl.handle.net/2246/3026

[B88] Emmons LH Leite YLR Kock D Costa LP (2002) A review of the named forms of *Phyllomys* (Rodentia: Echimyidae) with the description of a new species from coastal Brazil. American Museum Novitates 3380: 1–40. https://doi.org/10.1206/0003-0082(2002)380<0001:arotnf>2.0.co;2

[B89] Emmons LH (2015) Genus *Juscelinomys* Moojen, 1965. In: Patton JL, Pardiñas UFJ, D’Elía G (Eds) Mammals of South America (Vol. 2) – Rodents. The University of Chicago Press, Chicago, 225–228.

[B90] Feijó A Araujo A Fracasso MPA Santos KRP (2010) New records of three bat species for the Caatinga of the state of Paraíba, northeastern Brazil. Chiroptera Neotropical 16: 723–727.

[B91] Feijó A Garbino GST Campos BATP Rocha PA Ferrari SF Langguth A (2015a) Distribution of *Tolypeutes* Illiger, 1811 (Xenarthra: Cingulata) with comments on its biogeography and conservation. Zoological Science 32: 77–87. https://doi.org/10.2108/zs140186 10.2108/zs140186 25660700

[B92] Feijó A Langguth A (2013) Mamíferos de médio e grande porte do nordeste do Brasil: distribuição e taxonomia, com descrição de novas espécies. Revista Nordestina de Biologia 22(1/2): 3–225.

[B93] Feijó A Rocha PA Ferrari SF (2015b) How do we identify Micronycteris (Schizonycteris) sanborni Simmons, 1996 (Chiroptera, Phyllostomidae) reliably and where we can find this species in Brazil? Papéis Avulsos de Zoologia 55: 269–280. https://doi.org/10.1590/0031-1049.2015.55.20

[B94] Fernandes FA D’Andrea PS Bonvicino CR (2012) *Oligoryzomys stramineus* Bonvicino and Weksler, 1998 (Mammalia: Rodentia: Sigmodontinae): new records in northeastern Brazil. Check List 8(1): 184–186.

[B95] Fernandes-Ferreira H Gurgel-Filho NM Feijó A Mendonça SV Alves RRN Langguth A (2015) Non-volant mammals from Baturité Ridge, Ceará state, Northeast Brazil. Check List 11: 1630. https://doi.org/10.15560/11.3.1630

[B96] Ferreira RG Jerusalinsky L Silva TCF Fialho MS Roque AA Fernandes A Arruda F (2009) On the occurrence of *Cebus flavius* (Schreber 1774) in the Caatinga, and the use of semi-arid environments by *Cebus* species in the Brazilian state of Rio Grande do Norte. Primates 50: 357–362. https://doi.org/10.1007/s10329-009-0156-z 10.1007/s10329-009-0156-z 19575145

[B97] Fragaszy DM Liu Q Wright BW Allen A Brown CW et al. (2013) Wild bearded capuchin monkeys (*Sapajus libidinosus*) strategically place nuts in a stable position during nut-cracking. PLoS ONE 8(2): e56182. https://doi.org/10.1371/journal.pone.0056182 10.1371/journal.pone.0056182 PMC3584076 23460793

[B98] Fuzessy LF Silva IO Malukiewicz J Silva FFR Pônzio MC Boere V Ackermann RR (2014) Morphological variation in wild marmosets (*Callithrix penicillata* and *C. geoffroyi*) and their hybrids. Evolutionary Biology 41: 480. https://doi.org/10.1007/s11692-014-9284-5

[B99] Garbino GST (2015a) Defining genera of New World monkeys: the need for a critical view in a necessarily arbitrary task. International Journal of Primatology. https://doi.org/10.1007/s10764-015-9882-9

[B100] Garbino GST (2015b) How many marmoset (Primates: Cebidae: Callitrichinae) genera are there? A phylogenetic analysis based on multiple morphological systems. Cladistics 31: 652–678. https://doi.org/10.1111/cla.12106 10.1111/cla.12106 34753268

[B101] Gardner AL (2008 [2007]) Tribe Monodelphini Hershkovitz, 1992. In: Patton JL, Pardiñas UFJ, D’Elía G (Eds) Mammals of South America (Vol. 2) – Rodents. The University of Chicago Press, Chicago, 39–43.

[B102] Geise L Peresque R Sebastião H Shirai LT Astúa D Marroig G (2010) Non-volant mammals, Parque Nacional do Catimbau, Vale do Catimbau, Buíque, state of Pernambuco, Brazil, with karyologic data. Check List 6: 180–186. https://doi.org/10.15560/6.1.180

[B103] Geise L Hingst E Weksler ME Cerqueira R (1996) A new karyotype of *Calomys* (Rodentia: Sigmodontinae), with taxonomic considerations. Revista Brasileira de Genética 19(3): 45.

[B104] Giarla TC Voss RS Jansa SA (2010) Species limits and phylogenetic relationships in the didelphid marsupial genus *Thylamys* based on mitochondrial DNA sequences and morphology. Bulletin of the American Museum of Natural History 346: 1–67. https://doi.org/10.1206/716.1

[B105] Gomes LP Rocha CR Brandão RA Marinho-Filho J (2015) Mammal richness and diversity in Serra do Facão region, Southeastern Goiás state, central Brazil. Biota Neotropica 15: 1–11. http://dx.doi.org/10.1590/1676-0611-BN-2015-0033

[B106] Gonçalves PR Almeida FC Bonvicino CR (2005) A new species of *Wiedomys* (Rodentia: Sigmodontinae) from Brazilian Cerrado. Mammalian Biology 70: 46–60. https://doi.org/10.1078/1616-5047-00175

[B107] Gonçalves PR Myers P Vilela JF Oliveira JA (2006) Systematics of species of the genus *Akodon* (Rodentia: Sigmodontinae) in southeastern Brazil and implications for the biogeography of the campos de altitude. Miscellaneous Publications of the Museum of Zoology, University of Michigan 197: 1–26.

[B108] Giarla TC Voss RS Jansa SA (2010) Species limits and phylogenetic relationships in the didelphid marsupial genus *Thylamys* based on mitochondrial DNA sequences and morphology. Bulletin of the American Museum of Natural History Number 346:1–67. https://doi.org/10.1206/716.1

[B109] Gregorin R (2006) Taxonomia e variação geográfica das espécies do gênero *Alouatta* Lacépède (Primates, Atelidae) no Brasil. Revista Brasileira de Zoologia 23(1): 64–144. https://doi.org/10.1590/S0101-81752006000100005

[B110] Gregorin R Carmignotto AP Percequillo AR (2008) Quirópteros do Parque Nacional da Serra das Confusões, Piauí, nordeste do Brasil. Chiroptera Neotropical 14: 366–383.

[B111] Gregorin R Ditchfield AD (2005) New genus and species of nectar-feeding bat in the tribe Lonchophyllini (Phyllostomidae: Glossophaginae) from northeastern Brazil. Journal of Mammalogy 86(2): 403-414. https://doi.org/10.1644/BRB-229.1

[B112] Groves C (2001) Primate taxonomy. Smithsonian Institution Press, Washington DC, 350 pp.

[B113] Groves CP (2005) Family Atelidae In: Wilson DE, Reeder DM (Eds) Mammal Species of the World. A taxonomic and geographic reference (3^rd^ edn). The Johns Hopkins University Press, Baltimore, 148–151.

[B114] Gurgel-Filho NM Feijó A Langguth A (2015) Pequenos mamíferos do Ceará (marsupiais, morcegos e roedores sigmodontíneos) com discussão taxonômica de algumas espécies. Revista Nordestina de Biologia 23(2): 3–150. http://www.biblionline.ufpb.br/ojs/index.php/revnebio/article/view/25904

[B115] Gutiérrez EE Helgen KM (2013) Outdated taxonomy blocks conservation. Nature 495: 314. https://doi.org/10.1038/495314e 10.1038/495314e 23518556

[B116] Gutiérrez EE Anderson RP Voss RS Ochoa-G. J Aguilera M Jansa SA (2014) Phylogeography of the mouse opossum *Marmosa robinsoni*: insights into the biogeography of dry in northern South America. Journal of Mammalogy 95: 1175–1188. https://doi.org/10.1644/14-MAMM-A-069

[B117] Haag T Muschner VC Freitas LB Oliveira LFB Langguth AR Mattevi MS (2007) Phylogenetic relationships among species of the genus *Calomys* with emphasis on South American lowland taxa. Journal of Mammalogy 88: 769–776. https://doi.org/10.1644/05-MAMM-A-319R1.1

[B118] Hannibal W Neves-Godoi M (2015) Non-volant mammals of the Maracaju Mountains, southwestern Brazil: composition, richness and conservation. Revista Mexicana de Biodiversidad 86: 217–225. https://doi.org/10.7550/rmb.48618

[B119] Hegdal PL Blaskiewicz RW (1984) Evaluation of the potential hazard to Barn Owls of Talon (brodifacoum bait) used to control rats and house mice. Environmental Toxicology and Chemistry 3: 167–179. https://doi.org/10.1002/etc.5620030119

[B120] Hershkovitz P (1949) Mammals of northern Colombia. Preliminary report No. 4: Monkeys (Primates) with taxonomic revisions of some forms. Proceedings of the United States National Museum 98: 323–427. https://doi.org/10.5479/si.00963801.98-3232.323

[B121] Hershkovitz P (1955) Notes on American monkeys of the genus *Cebus* Journal of Mammalogy 36: 449–452. https://doi.org/10.2307/1375688

[B122] Hershkovitz P (1962) Evolution of Neotropical cricetine rodents (Muridae) with special reference to the phyllotine group. Fieldiana. Zoology 46: 1–524. https://doi.org/10.5962/bhl.title.2781

[B123] Hershkovitz P (1990a) The Brazilian rodent genus *Thalpomys* (Sigmodontinae, Cricetidae) with a description of a new species. Journal of Natural History 24: 763–783. https://doi.org/10.1080/00222939000770531

[B124] Hershkovitz P (1990b) Titis, new world monkeys of the genus *Callicebus* (Cebidae, Platyrrhini): a preliminary taxonomic review. Fieldiana Zoology 5: 1–109. 1993.

[B125] Hershkovitz P (1993) A new central Brazilian genus and species of sigmodontine rodent (Sigmodontinae) transitional between akodonts and oryzomyines, with a discussion of muroid molar morphology and evolution. Fieldiana Zoology 75: 1–18. https://doi.org/10.5962/bhl.title.3370

[B126] Hoffmann FG Lessa EP Smith MF (2002) Systematics of *Oxymycterus* with the description of a new species from Uruguay. Journal of Mammalogy 83: 408–420. https://doi.org/10.1644/1545-1542(2002)083<0408:SOOWDO>2.0.CO;2

[B127] Iack-Ximenes GE (2005) Sistemática de *Trinomys* Thomas, 1921 (Rodentia, Hystricognathi, Echimyidae). Ph.D. diss., Universidade de São Paulo, Departamento de Zoologia, São Paulo, Brazil. https://doi.org/10.1111/j.1523-1739.2005.00702.x

[B128] IBGE (Instituto Brasileiro de Geografia e Estatística) (2004a) Mapa de biomas do Brasil: escala 1:5.000.000. http://www.ibge.gov.br/ [Accessed on April 19, 2016]

[B129] IBGE (Instituto Brasileiro de Geografia e Estatística) (2004b) Mapa de Vegetação do Brasil. Available at http://www.ibge.gov.br/ [Accessed on April 19, 2016]

[B130] ICMBio-MMA (Instituto Chico Mendes, Ministério do Meio Ambiemte) (2016) Executive summary of Brazil red book of threatened species of fauna. Instituto Chico Mendes de Conservação da Biodiversidade. http://www.icmbio.gov.br/portal/images/stories/comunicacao/publicacoes/publicacoes-diversas/dcom_sumario_executivo_livro_vermelho_ed_2016.pdf [Accessed on December 19th, 2016]

[B131] Klink CA Machado RB (2005) Conservation of the Brazilian cerrado. Conservation Biology 19: 707–713.

[B132] Lacher T (2016) *Carterodon sulcidens* The IUCN Red List of Threatened Species 2016: e.T3921A14895982. http://www.iucnredlist.org/details/3921/0 [Downloaded 15 October 2016]

[B133] Leal ESB Silva DQ Ramalho DF Miller BG Filho PBP Neto JGP Guerra DQ Moura GJB Lyra-Neves RM Telino-Júnior WR (2013) Extension of the geographical distribution of *Lonchophylla dekeyseri* Taddei, Vizotto and Sazima, 1983 (Chiroptera: Phyllostomidae): new record in northeastern Brazil. Chiroptera Neotropical 19: 1220–1225.

[B134] Leal IR Silva JMC Tabarelli M Lacher TE Jr (2005) Changing the course of biodiversity conservation in the Caatinga of Northeastern Brazil. Conservation Biology 19: 701–706. https://doi.org/10.1111/j.1523-1739.2005.00703.x

[B135] Leal-Mesquita ER Yonenaga-Yassuda Y Chu TH Rocha PLB (1992) Chromosomal characterization and comparative cytogenetic analysis of two species of *Proechimys* (Echimyidae, Rodentia) from the Caatinga domain of state of Bahia, Brazil. Caryologia 45: 197–212. https://doi.org/10.1080/00087114.1992.10797223

[B136] Leite YLR (2003) Evolution and systematics of the Atlantic tree rats, genus *Phyllomys* (Rodentia, Echimyidae), with description of two new species. University of California Publications in Zoology 132: 1–118. https://doi.org/10.1525/california/9780520098497.001.0001

[B137] Leite YLR Loss AC (2015) Genus *Phyllomys* Lund, 1839. In: Patton JL, Pardiñas UFJ, D’Elía G (Eds) Mammals of South America (Vol. 2) – Rodents. The University of Chicago Press, Chicago, 915–928.

[B138] Leite YLR Patterson BD (2008) *Juscelinomys candango* The IUCN Red List of Threatened Species 2008: e.T10946A3228892. https://doi.org/10.2305/IUCN.UK.2008.RLTS.T10946A3228892.en [Downloaded 15 October 2016]

[B139] Lessa G Gonçalves PB Pessôa LM (2005) Variação geográfica em caracteres cranianos quantitativos de *Kerodon rupestris* (Wied, 1820) (Rodentia, Caviidae). Arquivos do Museu Nacional, Rio de Janeiro 63: 75–88. https://doi.org/10.1515/MAMM.2006.013

[B140] Locks M (1981) Nova espécie de *Oecomys* de Brasília, DF, Brasil (Cricetidae, Rodentia). Boletim do Museu Nacional, Rio de Janeiro 300: 1–7.

[B141] López-Baucells A Rocha R Mayés-García I Vulinec K Meyer CFJ (2013) First record of *Micronycteris sanborni* (Chiroptera: Phyllostomidae) from Central Amazonia, Brazil: range expansion and description of its echolocation. Mammalia 77: 1–6. https://doi.org/10.1515/mammalia-2013-0006

[B142] Loss C Leite Y (2016) *Phyllomys brasiliensis* The IUCN Red List of Threatened Species 2016: e.T6978A22209830. http://www.iucnredlist.org/details/6978/0 [Downloaded 15 October 2016]

[B143] Lynch-Alfaro JW Boubli JP Olson LE Fiore AD Wilson B Gutiérrez-Espeleta GA Chiou KL Schulte M Neitzel S Ross V Schwochow D Nguyen MTT Farias I Janson CH Alfaro ME (2012a) Explosive Pleistocene range expansion leads to widespread Amazonian sympatry between robust and gracile capuchin monkeys. Journal of Biogeography 39: 272–288. https://doi.org/10.1111/j.1365-2699.2011.02609.x

[B144] Lynch-Alfaro JW Silva JS Jr Rylands AB (2012b) How different are robust and gracile capuchin monkeys? An argument for the use of *Sapajus* and *Cebus* American Journal of Primatology 74:273–286. https://doi.org/10.1002/ajp.22007 10.1002/ajp.22007 22328205

[B145] Lynch-Alfaro JW Izar P Ferreira RG (2014) Capuchin monkey research priorities and urgent issues. American Journal of Primatology 76: 705–720. https://doi.org/10.1002/ajp.22269 10.1002/ajp.22269 24668460

[B146] Maia V Langguth A (1987) Chromosomes of the Brazilian rodent *Wiedomys pyrrhorhinos* (Wied, 1821). Revista Brasileira de Genética 10: 229–233.

[B147] Mares MA Braun JK Gettinger D (1989) Observations on the distribution and ecology of the mammals of the cerrado grasslands of central Brazil. Annals of the Carnegie Museum 58: 1–60.

[B148] Marinho-Filho J Guimarães MM Reis ML Rodrigues FHG Torres O Almeida G (1997) The discovery of the Brazilian three-banded armadillo in the cerrado of central Brazil. Edentata 3: 11–13.

[B149] Marinho-Filho J Langguth A (2016) *Wiedomys pyrrhorhinos* The IUCN Red List of Threatened Species 2008: e.T23076A9415389. https://doi.org/10.2305/IUCN.UK.2008.RLTS.T23076A9415389.en [Downloaded 15 October 2016]

[B150] Marinho-Filho J Rodrigues FHG Juarez KM (2002) The Cerrado mammals: diversity, ecology, and natural history.” In: Oliveira PS, Marquis RJ (Eds) The Cerrados of Brazil: Ecology and Natural History of a Neotropical Savanna. Columbia University Press, New York, 266–286. https://doi.org/10.7312/oliv12042-013

[B151] Marinho-Filho J Vieira E (2010) *Microakodontomys transitorius* The IUCN Red List of Threatened Species 2010: e.T136468A4295568. https://doi.org/10.2305/IUCN.UK.2010-2.RLTS.T136468A4295568.en [Downloaded 15 October 2016]

[B152] Marinho Filho J Bonvicino CR Vieira E (2016a) *Thalpomys cerradensis* The IUCN Red List of Threatened Species 2016: e.T21694A95402365. http://www.iucnredlist.org/details/21694/0 [Downloaded on 15 October 2016]

[B153] Marinho Filho J Bonvicino CR Vieira E (2016b) *Thalpomys lasiotis* The IUCN Red List of Threatened Species 2016: e.T21695A95402558. http://www.iucnredlist.org/details/21695/0 [Downloaded on 15 October 2016]

[B154] Marinho Filho J Veríssimo EW (1997) The rediscovery of *Callicebus personatus barbarabrownae* in northeastern Brazil with a new western limit to its distribution. Primates 38: 429–433. https://doi.org/10.1007/BF02381883

[B155] Marques EN Beltrão-Mendes R Ferrari SF (2013) Primates, Pitheciidae, *Callicebus barbarabrownae* Hershkovitz, 1990: new localities for the critically endangered titi monkey in the São Francisco basin, state of Sergipe, Brazil. Check List 9: 113–115. https://doi.org/10.15560/9.1.113

[B156] Martin PS Gheler-Costa C Lopes PC Rosalino LM Verdade LM (2012) Terrestrial non-volant small mammals in agro-silvicultural landscapes of Southeastern Brazil. Forest Ecology and Management 282: 185–195. https://doi.org/10.1016/j.foreco.2012.07.002

[B157] Mattevi MS Haag T Oliveira LFB Langguth AR (2005) Chromosome characterization of Brazilian species of *Calomys* Waterhouse, 1837 from Amazon, Cerrado and Pampa domains (Rodentia, Sigmodontinae). Arquivos do Museu Nacional, Rio de Janeiro 63(1): 175–181.

[B158] Mendes FDC Cardoso RM Ottoni EB Izar P Villar DNA Marquezan RF (2015) Diversity of nutcracking tool sites used by *Sapajus libidinosus* in Brazilian cerrado. American Journal of Primatology 77: 535–546. https://doi.org/10.1002/ajp.22373 10.1002/ajp.22373 25676549

[B159] Miles L Newton AC DeFries RS Ravilious C May I Blyth S Kapos V Gordon JE (2006) A global overview of the conservation status of tropical dry forests. Journal of Biogeography 33: 491–505. https://doi.org/10.1111/j.1365-2699.2005.01424.x

[B160] Miranda F Moraes-Barros N Superina M Abba AM (2014) *Tolypeutes tricinctus* The IUCN Red List of Threatened Species 2014: e.T21975A47443455 http://www.iucnredlist.org/details/21975/0 [Downloaded 15 October 2016]

[B161] Miranda GHB Faria DS (2001) Ecological aspects of black-pincelled marmoset (*Callithrix penicillata*) in the cerradão and dense cerrado of the Brazilian central plateau. Brazilian Journal of Biology 61: 397–404. https://doi.org/10.1590/S1519-69842001000300008 10.1590/s1519-69842001000300008 11706566

[B162] Miranda GB Oliveira LFB Andrades-Miranda J Langguth A Callegari-Jacques SM Mattevi MS (2009) Phylogenetic and phylogeographic patterns in sigmodontine rodents of the genus *Oligoryzomys* Journal of Heredity 100: 309–321. https://doi.org/10.1093/jhered/esn099 10.1093/jhered/esn099 19060234

[B163] MMA (Ministério do Meio Ambiente, República Federativa do Brasil) (2014) Lista nacional oficial [sic] de espécies da fauna ameaçada de extinção. Anexo I. Diário Oficial da União, seção 1. 245: 121–126. ISSN 1677-7042. Available at http://www.icmbio.gov.br/ [Downloaded 04 July 2016]

[B164] Moojen J (1943) Alguns mamíferos colecionados no nordeste do Brasil com a descricão de duas espécies novas e notas de campo. Boletim do Museu Nacional. Nova Série Zoolgia 5: 1–14.

[B165] Moojen J (1965) Nôvo gênero de Cricetidae do Brasil Central (Glires, Mammalia). Revista Brasileira de Biologia 25: 281–285.

[B166] Moojen J Locks M Langguth A (1997) A new species of *Kerodon*, Cuvier, 1825 from the State of Goiás, Brazil (Mammalia, Rodentia, Caviidae). Boletim do Museu Nacional, Nova Série Zoologia 377: 1–10.

[B167] Moratelli R Dias D (2015) A new species of nectar-feeding bat, genus *Lonchophylla*, from the Caatinga of Brazil (Chiroptera, Phyllostomidae). ZooKeys 514: 73–91. https://doi.org/10.3897/zookeys.514.10013 10.3897/zookeys.514.10013 PMC4525025 26261433

[B168] Nascimento FF Lazar A Menezes AN Durans ADM Moreira JC Salazar-Bravo J Andrea PSD Bonvicino CR (2013) The role of historical barriers in the diversification processes in open vegetation formations during the Miocene/Pliocene using an ancient rodent lineage as a model. PLoS ONE 8(4): e61924. https://doi.org/10.1371/journal.pone.0061924 10.1371/journal.pone.0061924 PMC3630152 24349576

[B169] do Nascimento MC Dias LH Gregorin R Lessa G (2013) Rediscovery of *Lonchophylla bokermanni* Sazima, Vizotto and Taddei, 1978 (Chiroptera: Phyllostomidae: Phyllostominae) in Minas Gerais, and new records for Espírito Santo, southeastern Brazil. Check List 9(5): 1046–1049. https://doi.org/10.15560/9.5.1046

[B170] Nimer E (1989) Climatologia do Brasil (2^nd^ edn). Instituto Brasileiro de Geografia e Estatística, Rio de Janeiro.

[B171] Nogueira MR Gregorin R Peracchi AL (2014a) Emended diagnosis of *Xeronycteris vieirai* (Mammalia: Chiroptera), with the first record of polyodontia for the genus. Zoologia 31: 175–180. https://doi.org/10.1590/S1984-46702014000200009

[B172] Nogueira MR Lima IP Moratelli R Tavares VC Gregorin R Peracchi AL (2014b) Checklist of Brazilian bats, with comments on original records. Check List 10: 808–821. https://doi.org/10.15560/10.4.808

[B173] Nogueira MR Peracchi AL Moratelli R (2007) Subfamília Phyllostominae. In: dos Reis NR, Peracchi AL, Pedro WA, Lima IP (Eds) Morcegos do Brasil. Londrina, Paraná, 61–97.

[B174] Nogueira MR Pol A Pessôa LM Oliveira JA Peracchi AL (2015) Small mammals (Chiroptera, Didelphimorphia, and Rodentia) from Jaíba, middle Rio São Francisco, northern Minas Gerais State, Brazil. Biota Neotropica 15(2): e20140126. https://doi.org/10.1590/1676-06032015012614

[B175] Nova MD Galbany J Górka K Pérez-Pérez A (2015) Taxonomic implications of molar morphology variability in capuchins. International Journal of Primatology. https://doi.org/10.1007/s10764-015-9850-4

[B176] Olifiers N Delciellos AC (2013) New record of *Lycalopex vetulus* (Carnivora, Canidae) in northeastern Brazil. Oecologia Australis 17(4): 533–537. https://doi.org/10.4257/oeco.2013.1704.08

[B177] Oliveira FF Langguth A (2004) Pequenos mamíferos (Didelphimorphia e Rodentia) de Paraíba e Pernambuco, Brasil. Revista Nordestina de Biologia 18: 19–86.

[B178] Oliveira JA Bonvicino CR (2006) Ordem Rodentia. In: dos Reis NR, Peracchi AL, Pedro WA, Lima IP (Eds) Mamíferos do Brasil. Londrina, Paraná, 347–406.

[B179] Oliveira JA Gonçalves PR (2015) Genus *Oxymycterus* Water house, 1837. In: Patton JL, Pardiñas UFJ, D’Elía G (Eds) Mammals of South America (Vol. 2) – Rodents. The University of Chicago Press, Chicago, 247–268.

[B180] Oliveira JA Gonçalves PR Bonvicino CR (2008) Mamíferos da Caatinga. In: Leal IR, Tabarelli M, Silva JMC (Eds) Ecologia e Conservação da Caatinga, 3rd edition. Editora Universitária, Universidade Federal de Pernambuco, Recife, 275–302.

[B181] Oliveira MM Kierulff MCM (2008) *Alouatta ululata* The IUCN Red List of Threatened Species 2008: e.T918A13094890. https://doi.org/10.2305/IUCN.UK.2008.RLTS.T918A13094890.en [Downloaded 15 October 2016]

[B182] Oliveira TG (1995) The Brazilian tree-banded armadillo *Tolypeutes tricinctus* in Maranhão. Edentata 2: 18–19.

[B183] Owen RD (2013) Ecology of small terrestrial mammals in an isolated cerrado patch, eastern Paraguay: communities, species, and effects of ENSO, precipitation, and fire. Mastozoología Neotropical 20(1): 97–112.

[B184] Paglia AP Fonseca GAB Rylands AB Herrmann G Aguiar LMS Chiarello AG Leite YLR Costa LP Siciliano S Kierulff MCM Mendes SL Tavares VC Mittermeier RA Patton JL (2012) Annotated Checklist of Brazilian Mammals. 2nd Edition. Occasional Papers in Conservation Biology 6: 1–76. http://www.conservation.org.br/publicacoes/ files/annotated_checklist_of_brazilian_mammals_2nd_edition.pdf

[B185] Pardiñas UFJ Bezerra AMR (2015) Genus *Gyldenstolpia* Pardiñas, D’Elía, and Teta, 2009. In: Patton JL, Pardiñas UFJ, D’Elía G (Eds) Mammals of South America (Vol. 2) – Rodents. The University of Chicago Press, Chicago, 222–225.

[B186] Pardiñas UFJ Lessa G Teta P Salazar-Bravo J Câmara EMVC (2014) A new genus of sigmodontine rodent from eastern Brazil and the origin of the tribe Phyllotini Journal of Mammalogy 95: 201–215. https://doi.org/10.1644/13-MAMM-A-208

[B187] Pardiñas UFJ Teta P (2015) Genus *Thalpomys* Thomas, 1916. In: Patton JL, Pardiñas UFJ, D’Elía G (Eds) Mammals of South America (Vol. 2) – Rodents. The University of Chicago Press, Chicago, 274–277.

[B188] Pardiñas UFJ Teta P Alvarado-Serrano D Geise L Jayat PJ Ortiz PE Gonçalves PR D’Elia G (2015) Genus *Akodon* Meyen, 1833. In: Patton JL, Pardiñas UFJ, D’Elía G (Eds) Mammals of South America (Vol. 2) – Rodents. The University of Chicago Press, Chicago, 144–204.

[B189] Patterson BD D’Elia G Pardinas U Teta P (2008) *Oxymycterus delator* The IUCN Red List of Threatened Species 2008: e.T15784A5156986. https://doi.org/10.2305/IUCN.UK.2008.RLTS.T15784A5156986.en [Downloaded 15 October 2016]

[B190] Patton J Catzeflis F Weksler M Percequillo M (2008a) *Rhipidomys cariri* The IUCN Red List of Threatened Species 2008: e.T136357A4279986. https://doi.org/10.2305/IUCN.UK.2008.RLTS.T136357A4279986.en [Downloaded 15 October 2016]

[B191] Patton JL Emmons LH (2015) Family Dasyproctidae Bonaparte, 1838. In: Patton JL, Pardiñas UFJ, D’Elía G (Eds) Mammals of South America (Vol. 2) – Rodents. The University of Chicago Press, Chicago, 733–762.

[B192] Patton JL Pardiñas UFJ D’Elía G (Eds) (2015) Mammals of South America (Vol. 2) – Rodents. The University of Chicago Press. Chicago, Illinois.

[B193] Pennington RT Lewis GP Ratter JA (2006) Neotropical savannas and seasonally dry forests: plant diversity, biogeography and conservation. CRC Press Taylor & Francis Group, New York. https://doi.org/10.1201/9781420004496

[B194] Pereira LG Geise L (2009) Non-flying mammals of Chapada Diamantina (Bahia, Brazil). Biota Neotropica 9: 185–196. https://doi.org/10.1590/S1676-06032009000300019

[B195] Percequillo AR (2015) Genus *Cerradomys* Weksler, Percequillo, and Voss, 2006. In: Patton JL, Pardiñas UFJ, D’Elía G (Eds) Mammals of South America (Vol. 2) – Rodents. The University of Chicago Press, Chicago, 300–308.

[B196] Percequillo A Weksler M (2008) *Euryoryzomys lamia* The IUCN Red List of Threatened Species 2008: e.T15602A4890429. https://doi.org/10.2305/IUCN.UK.2008.RLTS.T15602A4890429.en [Downloaded 15 October 2016]

[B197] Paresque R Hanson JD (2015) Genus *Microakodontomys* Hershkovitz, 1993. In: Patton JL, Pardiñas UFJ, D’Elía G (Eds) Mammals of South America (Vol. 2) – Rodents. The University of Chicago Press, Chicago, 354.

[B198] Pessôa LM Tavares WC Neves ACA da Silva ALG (2015a) Genus *Thrichomys* E.- L. Trouessart, 1880. In: Patton JL, Pardiñas UFJ, D’Elía G (Eds) Mammals of South America (Vol. 2) – Rodents. The University of Chicago Press, Chicago, 989–999.

[B199] Pessôa LM Tavares WC de Oliveira JA Patton JL (2015b) Genus *Trinomys* Thomas, 1921. In: Patton JL, Pardiñas UFJ, D’Elía G (Eds) Mammals of South America (Vol. 2) – Rodents. The University of Chicago Press, Chicago, 999–119.

[B200] Pessôa LM Oliveira-Corrêa MM Oliveira JA Lopes MOG (2004) Karyological and morphometric variation in the genus *Thrichomys* (Rodentia: Echimyidae). Mammalian Biology 69: 258–269. https://doi.org/10.1078/1616-5047-00141

[B201] Pessôa LM Strauss RE (1999) Cranial size and shape variation, pelage and bacular morphology, and subspecific differentiation in spiny rats, *Proechimys albispinus* (I. Geoffroy, 1838), from northeastern Brazil. Bonner Zoologische Beiträge 48: 231–243.

[B202] Pontes ARM Malta A Asfora PH (2006) A new species of capuchin monkey, genus *Cebus* Erxleben (Cebidae, Primates) found at the very brink of extinction in the Pernambuco Endemism Centre. Zootaxa 1200: 1–12.

[B203] Prado DE (2008) As Caatingas da América do Sul. In: Leal IR, Tabarelli M, Silva JMC (Eds) Ecologia e conservação da Caatinga, 3rd ed. Editora Universitária, Universidade Federal de Pernambuco, Recife, 3–73.

[B204] Prado JR Percequillo AR (2013) On the geographic distribution of the genera of tribe Oryzomyini on South America, with some comments on the patterns of diversity and Reig’s areas of original differentiation. Arquivos de Zoologia 44: 1–124. https://doi.org/10.11606/issn.2176-7793.v44i1p1-120

[B205] Printes RC Jerusalinsky L Sousa MC Rodrigues LRR Hirsch A (2013) Zoogeography, genetic variation and conservation of the *Callicebus personatus* group. In: Veiga LM, Barnett AA, Ferrari SF, Norconk MA (Eds) Evolutionary biology and conservation of Titis, Sakis and Uacaris. Cambridge University Press, New York, 43–49. https://doi.org/10.1017/CBO9781139034210.050

[B206] Printes RC Rylands AB Bicca-Marques JC (2011) Distribution and status of the critically endangered blond titi monkey *Callicebus barbarabrownae* of north-east Brazil. Oryx 45: 439–443. https://doi.org/10.1017/S0030605311000111

[B207] dos Reis SF Pessôa LM (1995) *Proechimys albispinus minor*, a new subspecies from the state of Bahia, northeastern Brazil (Rodentia: Echimyidae). Zeitschrift für Säugetierkunde 60: 181–190.

[B208] Ribeiro R Bezerra AMR Marinho-Filho JS (2010) Coleções científicas e conservação de mamíferos do Cerrado. Pp. 413–436. In: Diniz IR, Marinho-Filho JS, Machado RB, Cavalcanti R (Eds) Conhecimento científico quantitativo como subsídio para ações de conservação. Thesaurus. Brasília, DF.

[B209] Ribeiro R Rocha CR Marinho-Filho J (2011) Natural history and demography of *Thalpomys lasiotis* (Thomas, 1916), a rare and endemic species from Brazilian savanna. Acta Theriologica 56: 275–282. https://doi.org/10.1007/s13364-011-0026-0

[B210] Roach N (2016) *Kerodon acrobata* The IUCN Red List of Threatened Species 2016: e.T136222A22190183. http://www.iucnredlist.org/details/136222/0 [Downloaded 15 October 2016]

[B211] Roach N Naylor L (2016a) *Trinomys yonenagae* The IUCN Red List of Threatened Species 2016: e.T136414A22212204. http://www.iucnredlist.org/details/136414/0 [Downloaded 15 October 2016]

[B212] Roach N Naylor L (2016b) *Thrichomys apereoides* The IUCN Red List of Threatened Species 2016: e.T21839A22206242 http://www.iucnredlist.org/details/21839/0 [Downloaded 15 October 2016]

[B213] Rocha PLB (1995 [1996]) *Proechimys yonenagae*, a new species of spiny rat (Rodentia: Echimyidae) from fossil sand dunes in the Brazilian Caatinga. Mammalia 59: 537–549. https://doi.org/10.1515/mamm.1995.59.4.537

[B214] Rocha CR Ribeiro R Takahashi FSC Marinho-Filho J (2011a) Microhabitat use by rodent species in a central Brazilian cerrado. Mammalian Biology 76: 651–653. https://doi.org/10.1016/j.mambio.2011.06.006

[B215] Rocha RG Ferreira E Costa BMA Martins ICM Leite YLR Costa LP Fonseca C (2011b) Small mammals of the mid-Araguaia River in central Brazil, with the description of a new species of climbing rat. Zootaxa 2789: 1–34.

[B216] Rocha RG Fonseca C Zhou Z Leite YLR Costa LAP (2012) Taxonomic and conservation status of the elusive *Oecomys cleberi* (Rodentia, Sigmodontinae) from central Brazil. Mammalian Biology 77: 414–419 https://doi.org/10.1016/j.mambio.2012.02.004

[B217] Rosenberger AL (2012) New World monkey nightmares: science, art, use, and abuse (?) in platyrrhine taxonomic nomenclature. American Journal of Primatology 74: 692–695. https://doi.org/10.1002/ajp.22037 10.1002/ajp.22037 22605529

[B218] Rosenberger AL Matthews LJ (2008) *Oreonax*–not a genus. Neotropical Primates 15: 8–12. https://doi.org/10.1896/044.015.0102

[B219] Rylands AB Coimbra-Filho AF Mittermeier RA (2009) The systematics and distributions of the Marmosets (*Callithrix*, *Callibella*, *Cebuella*, and *Mico*) and *Callimico* (Callimico) (Callitrichidae, Primates). In Ford SM, Porter LM, Davis LC (Eds) The Smallest Anthropoids The Marmoset/Callimico Radiation. Springer, US, 25–61. https://doi.org/10.1007/978-1-4419-0293-1

[B220] Rylands AB Heymann EW Lynch Alfaro J Buckner JC Roos C Matauschek C Boubli JP Sampaio R Mittermeier RA (2016) Taxonomic review of the New World tamarins (Primates: Callitrichidae). Zoological Journal of the Linnean Society 177: 1003–1028. https://doi.org/10.1111/zoj.12386

[B221] Rylands AB Kierulff MCM (2015) *Sapajus libidinosus* The IUCN Red List of Threatened Species 2015: e.T136346A70613080. https://doi.org/10.2305/IUCN.UK.2015-1.RLTS.T136346A70613080.en [Downloaded 15 October 2016]

[B222] Rylands AB Mendes SL (2008) *Callithrix penicillata* The IUCN Red List of Threatened Species 2008: e.T41519A10486326. https://doi.org/10.2305/IUCN.UK.2008.RLTS.T41519A10486326.en [Downloaded 15 October 2016]

[B223] Sá-Neto RJ Marinho-Filho J (2013) Bats in fragments of xeric woodland caatinga in Brazilian semiarid. Journal of Arid Environments 90: 88–94. https://doi.org/10.1016/j.jaridenv.2012.10.007

[B224] Salazar-Bravo J (2015) Genus *Calomys* Water house, 1837. In: Patton JL, Pardiñas UFJ, D’Elía G (Eds) Mammals of South America (Vol. 2) – Rodents. The University of Chicago Press, Chicago, 481–507.

[B225] Sampaio EVSB (1995) Overview of the Brazilian Caatinga. In: Bullock SH, Mooney HA, Medina E (Eds) Seasonally dry tropical forests. Cambridge University Press, Cambridge, 35–63. https://doi.org/10.1017/CBO9780511753398.003

[B226] Sanborn CC (1930) Distribution and habits of the three-banded armadillo *Tolypeutes* Journal of Mammalogy 11: 61–68. https://doi.org/10.2307/1373787

[B227] de la Sancha NU D’Elía G (2015) Additions to the Paraguayan mammal fauna: the first records of two marsupials (Didelphimorphia, Didelphidae) with comments on the alpha taxonomy of *Cryptonanus* and *Philander* Mammalia 79: 343–356. https://doi.org/10.1515/mammalia-2013-0176

[B228] de la Sancha NU D’Elía G Tribe CJ Perez PE Valdez L Pine RH (2011) *Rhipidomys* (Rodentia, Cricetidae) from Paraguay: noteworthy new records and identity of the Paraguayan species. Mammalia 75: 269–276. https://doi.org/10.1515/mamm.2011.022

[B229] Santos CF Nogueira MR Cunha NL Carvalho LFAC Fischer E (2010) Southernmost record of the Sanborn’s big-eared bat, *Micronycteris sanborni* (Chiroptera, Phyllostomidae). Mammalia 74: 457–460. https://doi.org/10.1515/MAMM.2010.041

[B230] Santos IB Fonseca GAB Rigueira SE Machado RB (1994) The rediscovery of the Brazilian three banded armadillo and notes on its conservation status. Edentata 1: 11–15.

[B231] Sampaio EVSB (1995) Overview of the Brazilian Caatinga. In: Bullock SH, Mooney HA, Medina E (Eds) Seasonally Dry Tropical Forests. Cambridge University Press, Cambridge, 35–63. https://doi.org/10.1017/CBO9780511753398.003

[B232] Sazima I Vizotto LD Taddei VA (1978) Uma nova espécie de *Lonchophylla* da Serra do Cipó, Minas Gerais, Brasil (Mammalia, Chiroptera, Phyllostomidae). Revista Brasileira de Biologia 38: 81–89.

[B233] Siles L Brooks DM Aranibar H Tarifa T Vargas RJM Rojas JM Baker RJ (2013) A new species of *Micronycteris* (Chiroptera: Phyllostomidae) from Bolivia. Journal of Mammalogy 94: 881–896. https://doi.org/10.1644/12-MAMM-A-259.1

[B234] Silva CJM Oren DC (1993) Observations on the habitat and distribution of the Brazilian three banded armadillo *Tolypeutes tricinctus*, a threatened Caatinga endemic. Mammalia 57: 149–151.

[B235] Silva JMC da Bates JM (2002) Biogeographic patterns and conservation in the South American Cerrado: a tropical savanna hotspot. Bioscience 52: 225–234. https://doi.org/10.1641/0006-3568(2002)052[0225:BPACIT]2.0.CO;2

[B236] Silva SSP Dias D Martins MA Guedes PG Almeida JC Cruz AP Serra-Freire NM Damascena JS Peracchi AL (2015) Bats (Mammalia: Chiroptera) from the Caatinga scrublands of the Crateus region, northeastern Brazil, with new records for the state of Ceará. Mastozoología Neotropical 22: 335–348.

[B237] Silva-Junior JS (2001) Especiação nos macacos-prego e caiararas, gênero *Cebus* Erxleben, 1777 (Primates, Cebidae). Ph.D. Thesis, Universidade Federal do Rio de Janeiro, Rio de Janeiro.

[B238] Simmons NB (1996) A new species of *Micronycteris* (Chiroptera: Phyllostomidae) from northeastern Brazil, with comments on phylogenetic relationships. American Museum Novitates 3158: 1–34.

[B239] Solari S (2015) *Xeronycteris vieirai* The IUCN Red List of Threatened Species 2015: e.T136321A22021092. https://doi.org/10.2305/IUCN.UK.2015-4.RLTS.T136321A22021092.en [Downloaded 15 October 2016]

[B240] Souza DP Asfora PH Lira TC Astúa D (2010) Small mammals in Barn Owl (*Tyto alba*–Aves, Strigiformes) pellets from Northeastern Brazil, with new records of *Gracilinanus* and *Cryptonanus* (Didelphimorphia, Didelphidae). Mammalian Biology 75: 370–374. https://doi.org/10.1016/j.mambio.2009.08.003

[B241] Souza ALG (2011) Sistemática integrativa e abordagem biogeográfica de linhagens de roedores da Caatinga. Ph.D. diss., Museu Nacional, Universida de Federal do Rio de Janeiro, Brazil.

[B242] Souza ALG Corrêa MMO Aguilar CT Pessôa LM (2011a) A new karyotype of *Wiedomys pyrrhorhinus* (Rodentia: Sigmodontinae) from Chapada Diamantina, northeastern Brazil. Zoologia 28: 92–96. https://doi.org/10.1590/S1984-46702011000100013

[B243] Souza ALG Corrêa MMO Pessôa LM (2006) Morphometric discrimination between *Trinomys albispinus* (I. Geoffroy, 1838) and *Trinomys minor* (Reis & Pessôa, 1995) from Chapada Diamantina, Bahia, Brazil, and the karyotype of *Trinomys albispinus* (Rodentia, Echimyidae). Arquivos do Museu Nacional, Rio de Janeiro 64(4): 325–332.

[B244] Stumpp R Nascimento-Costa MC Boroni NL Duarte TS Lessa G (2016) Contributions to the knowledge of small mammals (Mammalia) from northwestern Minas Gerais, Brazil. Boletim do Museu de Biologia Mello Leitão, nova série 38(1): 1–21.

[B245] Taddei VA Lim BK (2010) A new species of *Chiroderma* (Chiroptera, Phyllostomidae) from Northeastern Brazil. Brazilian Journal of Biology 70: 381–386. 10.1590/s1519-69842010000200021 20549066

[B246] Taddei VA Vizotto LD Sazima I (1983) Uma nova espécie de *Lonchophylla* do Brasil e chave para identificação das espécies do gênero (Chiroptera, Phyllostomidae). Ciência e Cultura 35: 625–629.

[B247] Tavares V Aguirre L (2008) *Micronycteris sanborni* The IUCN Red List of Threatened Species 2008: e.T40029A10308158. https://doi.org/10.2305/IUCN.UK.2008.RLTS.T40029A10308158.en [Downloaded 15 October 2016]

[B248] Tavares WC Pessôa LM Seuánez HN (2016) Stability and acceleration of phenotypic evolution in spiny rats (*Trinomys*, Echimyidae) across different environments. Zoological Journal of the Linnean Society 178: 149–162. https://doi.org/10.1111/zoj.12406

[B249] Teta P Cañon C Patterson BD Pardiñas UFJ (2016) Phylogeny of the tribe Abrotrichini (Cricetidae, Sigmodontinae): integrating morphological and molecular evidence into a new classification. Cladistics. [Early View] https://doi.org/10.1111/cla.12164 10.1111/cla.12164 34710969

[B250] Teixeira TSM Dias D Vale MM (2014) New records and a taxonomic review prompts reassessment of *Lonchophylla bokermanni*, a rare bat endemic to the Brazilian Cerrado. Oryx 49: 71–73. https://doi.org/10.1017/S0030605314000192

[B251] Tribe CJ (2005) A new species of *Rhipidomys* (Rodentia, Muroidea) from north-eastern Brazil. Arquivos do Museu Nacional, Rio de Janeiro 63: 131–146.

[B252] Tribe CJ (2015) Genus *Rhipidomys* Tschudi, 1845 In: Patton JL, Pardiñas UFJ, D’Elía G (Eds) Mammals of South America (Vol. 2) – Rodents. The University of Chicago Press, Chicago, 583–617.

[B253] Veiga LM Printes RC Rylands AB Kierulff CM de Oliveira MM Mendes SL (2008) *Callicebus barbarabrownae* The IUCN Red List of Threatened Species 2008: e.T39929A10291470. https://doi.org/10.2305/IUCN.UK.2008.RLTS.T39929A10291470.en [Downloaded 15 October 2016]

[B254] Viana MC Bonvicino CR Ferreira JG Jerusalinsky L Langguth A Seuánez H (2015) Understanding the relationship between *Alouatta ululata* and *Alouatta belzebul* (Primates: Atelidae) based on cytogenetics and molecular phylogeny. Oecologia Australis 19: 173–182. https://doi.org/10.4257/oeco.2015.1901.11

[B255] Vieira EM Palma ART (1996) Natural history of *Thylamys velutinus* (Marsupialia, Didelphidae) in central Brazil. Mammalia 60: 481–484. https://doi.org/10.1515/mamm-1996-0313

[B256] Vilela AA Del-Claro K (2011) Feeding behavior of the black-tufted-ear marmoset (*Callithrix penicillata*) (Primata, Callitrichidae) in a tropical cerrado savanna. Sociobiology 58: 1–6.

[B257] Vilela SL (2007) Sympatry and diet of *Callithrix penicillata* (Hershkovitz) (Callitrichidae) and *Cebus libidinosus* (Spix) (Cebidae) in gallery forests from Distrito Federal, Brasil. Revista Brasileira de Zoologia 24(3): 601–607. https://doi.org/10.1590/S0101-81752007000300012

[B258] de Vivo M (1991) Taxonomia de *Callithrix* Erxleben, 1777 (Callitrichidae Primates). Fundacão Biodiversitas, Belo Horizonte, 105 pp.

[B259] de Vivo M Carmignotto AP (2015) Family Sciuridae G. Fischer, 1817. In: Patton JL, Pardiñas UFJ, D’Elía G (Eds) Mammals of South America (Vol. 2) – Rodents. The University of Chicago Press, Chicago, 1–48.

[B260] Voss RS (2015a) Genus *Pseudoryzomys* Hershkovitz, 1962. In: Patton JL, Pardiñas UFJ, D’Elía G (Eds) Mammals of South America (Vol. 2) – Rodents. The University of Chicago Press, Chicago, 443–445.

[B261] Voss RS (2015b) Family Erethizontidae Bonaparte, 1845. In: Patton JL, Pardiñas UFJ, D’Elía G (Eds) Mammals of South America (Vol. 2) – Rodents. The University of Chicago Press, Chicago, 786–805.

[B262] Voss RS Gutiérrez EE Solari S Rossi RV Jansa SA (2014) Phylogenetic relationships of mouse opossums (Didelphidae, *Marmosa*) with a revised subgeneric classification and notes on sympatric diversity. American Museum Novitates 3817: 1–27. https://doi.org/10.1206/3817.1

[B263] Voss RS Lunde DP Jansa SA (2005) On the contents of *Gracilinanus* Gardner and Creighton, 1989, with the description of a previously unrecognized clade of small didelphid marsupials. American Museum Novitates 3482: 1–34. https://doi.org/10.1206/0003-0082(2005)482[0001:OTCOGG]2.0.CO;2

[B264] Voss RS Myers P Catzeflis F Carmignotto AP Barreiro J (2009) The six opossums of Félix de Azara: identification, taxonomic history, neotype designations, and nomenclatural recommendations. In: Voss RS, Carleton MD (Eds) Systematic mammalogy: contributions in honor of Guy G. Musser. Bulletin of the American Museum of Natural History 331: 406–433. https://doi.org/10.1206/582-11.1

[B265] Weksler M Bonvicino CR (2005) Taxonomy of pigmy rice rats (genus *Oligoryzomys*, Rodentia: Sigmodontinae) of the Brazilian Cerrado, with the description of two new species. Arquivos do Museu Nacional, Rio de Janeiro 63: 113–130.

[B266] Weksler M Bonvicino CR (2008a) *Galea flavidens* The IUCN Red List of Threatened Species 2008: e.T8823A12934467. https://doi.org/10.2305/IUCN.UK.2008.RLTS.T8823A12934467.en [Downloaded 15 October 2016]

[B267] Weksler M Bonvicino CR (2008b) *Oligoryzomys moojeni* The IUCN Red List of Threatened Species 2008: e.T136336A4276271. https://doi.org/10.2305/IUCN.UK.2008.RLTS.T136336A4276271.en [Downloaded 15 October 2016]

[B268] Weksler M Bonvicino CR (2008c) *Oligoryzomys rupestris* The IUCN Red List of Threatened Species 2008: e.T136425A4289978. http://dx.doi.org/10.2305/IUCN.UK.2008.RLTS.T136425A4289978.en [Downloaded 15 October 2016]

[B269] Weksler M Bonvicino CR (2008d) *Oligoryzomys stramineus* The IUCN Red List of Threatened Species 2008: e.T29418A9495706. https://doi.org/10.2305/IUCN.UK.2008.RLTS.T29418A9495706.en [Downloaded 15 October 2016]

[B270] Werneck FP (2011) The diversification of eastern South American open vegetation biomes: historical biogeography and perspectives. Quaternary Science Reviews 30: 1630–1648. https://doi.org/10.1016/j.quascirev.2011.03.009

[B271] Wetzel RM Gardner AL Redford KH Eisenberg JF (2007 [2008]) Order Cingulata Illiger, 1811. In: Patton JL, Pardiñas UFJ, D’Elía G (Eds) Mammals of South America (Vol. 2) – Rodents. The University of Chicago Press, Chicago, 128–157.

[B272] Winge H (1887 [1888]) Jordfunde og nulevende Gnavere (Rodentia) fra Lagoa Santa, Minas Geraes, Brasilien: med udsigt over gnavernes indbyrdes slaegtskab. E Museo Lundii, Kjöbenhavn 1(3): 1–178. [Cited as either 1887 or 1888; handwritten year of publication given as “1887 (88)” in digital copy available on at http://www.biodiversitylibrary.org/]

[B273] Woodman N Timm RM (2006) Characters and phylogenetic relationships of nectar-feeding bats, with descriptions of new *Lonchophylla* from western South America (Mammalia: Chiroptera: Phyllostomidae: Lonchophyllini). Proceedings of the Biological Society of Washington 119: 437–476. https://doi.org/10.2988/0006-324X(2006)119[437:CAPRON]2.0.CO;2

[B274] Zappes IA Portella AS Lessa GM (2014) Description of karyotype of *Kerodon acrobata*, an endemic rodent in Brazilian Cerrado. Brazilian Journal of Biology 74(1): 251–256. https://doi.org/10.1590/1519-6984.23512 10.1590/1519-6984.23512 25055111

[B275] Zimbres BQC de Aquino PPU Machado RB Silveira L Jácomo ATA Sollmann R Tôrres NM Furtado MM Marinho-Filho J (2012) Range shifts under climate change and the role of protected areas for armadillos and anteaters. Biological Conservation 152: 53–61. https://doi.org/10.1016/j.biocon.2012.04.010

[B276] Zimbres B Furtado MM Jácomo ATA Silveira L Sollmann R Tôrres NM Machado RB Marinho-Filho J (2013) The impact of habitat fragmentation on the ecology of xenarthrans (Mammalia) in the Brazilian Cerrado. Landscape Ecology 28: 259–269 https://doi.org/10.1007/s10980-012-9832-2

